# Evaluating a neural-efficiency account of the association between motor competence and attentional stability in preschoolers: An fNIRS study

**DOI:** 10.1016/j.dcn.2026.101787

**Published:** 2026-07-22

**Authors:** Yang You, Bingqi Fu, Haoran Mao, Lulu Cheng, Yang Li, Zeyu Sun

**Affiliations:** a China University of Petroleum (East China), China; b Qingdao University Affiliated Qingdao Women and Children’s Hospital, China; c Heping District Teachers’ Training School, China

**Keywords:** Motor development, Attentional stability, Neural efficiency, Prefrontal cortex (PFC), fNIRS, Embodied cognition

## Abstract

Motor development and attentional control are closely intertwined during early childhood, yet the neural basis of this association remains insufficiently understood. Guided by an embodied-cognition framework, the present study examined the association between motor competence and task-based attentional stability and tested whether behavioral differences were accompanied by differences in prefrontal oxygenated hemoglobin (HbO) responses. Sixty-two typically developing children aged 3–6 years completed the motor assessment and cancellation task. Behavioral analyses included all 62 children, whereas usable fNIRS data were available for 61 children after quality control. The results showed that (1) at the behavioral level, higher motor competence was associated with greater attentional stability; the descriptive subgroup pattern appeared more pronounced in the late-preschool group, although the age group × motor-competence level interaction was not statistically significant; and (2) at the neural level, none of the reported ROI regression coefficients for age group, motor-competence level, or their interaction reached statistical significance. Complementary channel-wise analyses revealed different suprathreshold activation patterns across age and motor-competence groups; however, these exploratory patterns did not provide direct evidence of spatial focalization or neural efficiency. The findings are therefore compatible with, but do not establish, a neural-efficiency account. By integrating behavioral and neuroimaging measures, the study provides evidence of an association between motor competence and task-based attentional stability.

## 1 Introduction

Motor development during early childhood is a critical foundation for cognitive functions, particularly attention and executive control. As children age, their ability to maintain focus, inhibit distractions, and flexibly shift attention develops progressively, with these skills serving as essential building blocks for later cognitive performance (Best and Miller, 2010). Recent evidence suggests a strong link between motor development and cognitive abilities, particularly in the domains of executive functions and attentional regulation (Anderson, 2002, Wassenberg et al., 2005). Both gross and fine motor skills have been shown to predict a child’s ability to sustain attention and control impulses, implying that the maturation of motor skills may support the emergence of self-regulation and attentional processes (Tseng et al., 2004). In the present study, we explicitly distinguish between related but non-equivalent constructs to improve conceptual precision. “Motor development” refers to the age-related maturational progression of motor systems across early childhood (Adolph and Franchak, 2017, Faber et al., 2024), whereas “motor competence” (or motor ability) denotes an individual’s current level of performance as indexed by standardized assessments (Burton and Rodgerson, 2001, Stodden et al., 2008). This distinction allows developmental stage (age-related change) to be separated from individual variability in motor proficiency. Similarly, “attentional control” is used to denote a broad executive function responsible for the allocation and regulation of cognitive resources (Diamond, 2013, Petersen and Posner, 2012), whereas “attentional stability” refers to a more specific behavioral construct capturing the ability to sustain attention and resist performance fluctuations over time (Esterman et al., 2013, Tamm et al., 2012). In the present study, attentional stability serves as the primary operational measure of attention during task performance.

This interplay between motor and cognitive development is rooted in the concept of embodied cognition, which suggests that cognitive processes are shaped through interactions between bodily experiences and neural systems (McClelland and Tominey, 2015). Early sensorimotor experience may contribute to the integration of processes supporting attention and executive control (Diamond, 2000, Gottwald et al., 2016). From this perspective, motor and attentional systems are not independent developmental domains but dynamically coupled components of a unified neurocognitive system. Mechanistically, this coupling can be grounded in established neurodevelopmental architectures. Complex motor execution and top-down attentional control are supported by partially overlapping neural circuits, including cerebellar–cortical loops and frontoparietal control networks, which are further modulated by dopaminergic pathways (Diamond, 2000, Stoodley et al., 2012). These shared systems provide a plausible neural substrate through which motor experience may shape attentional control during early development.

Despite these insights, the neural association underlying the relationship between motor development and attentional stability, particularly in preschool-aged children, remains poorly understood. Critically, prior research has largely treated prefrontal activation as a unidimensional indicator of cognitive engagement, implicitly assuming that better performance is associated with increased neural recruitment. However, this assumption is theoretically under-specified and has rarely been directly tested in early childhood populations.

What remains unknown in the current literature is not whether motor competence relates to attention, but how individual differences in motor competence are instantiated at the neural level. Specifically, it is unclear whether developmental improvements in attentional stability are supported by increased metabolic demand in the prefrontal cortex (resource-recruitment account) or by more efficient and optimized neural processing (neural-efficiency account). This distinction reflects fundamentally different models of brain-behavior coupling and has important implications for understanding early cognitive development.

Higher prefrontal activation does not necessarily indicate better performance, particularly when greater activation reflects compensatory control demands. In different age groups, PFC activation may represent different control demands (Surkar et al., 2021). For younger children, PFC activation may reflect early compensatory control, where the brain needs to allocate more resources to support motor control and attentional regulation systems that are still maturing. As a result, PFC activation might be higher in these children, compensating for the underdeveloped neural systems. In contrast, for older children, as motor and attentional systems become more efficient, PFC activation may decrease, reflecting more optimized brain activity, rather than compensatory processes. However, interpreting stable oxygenated hemoglobin (HbO) activation as evidence of neural efficiency requires careful consideration, as alternative explanations such as task-invariance, measurement insensitivity, or physiological ceiling effects cannot be ruled out without an explicit theoretical framework.

To improve theoretical specificity, the present study situates the proposed motor-attention coupling framework within established neurodevelopmental systems. Motor-related processes are closely linked to cerebellar-cortical loops, which support coordination and prediction, and to frontoparietal control networks, which are central to attentional regulation and executive control. These systems are further modulated by dopaminergic pathways involved in cognitive stability and flexibility. Within this framework, motor competence is hypothesized to be associated with more efficient integration across these systems, which may in turn relate to more stable attentional performance.

Importantly, the present study distinguishes between three levels of explanation. First, behavioral coupling refers to the association between motor competence and attentional stability. Second, neurobehavioral correspondence refers here to whether behavioral group differences are accompanied by differences in neural activation; individual-level performance–activation coupling was not directly tested. Third, neural efficiency accounts refer to changes in how neural resources are organized and utilized, rather than changes in overall activation magnitude. This distinction allows the current study to move beyond descriptive associations toward a more structured and testable framework.

To empirically test these predictions, the present study integrates standardized behavioral assessments of motor development with concurrent functional near-infrared spectroscopy (fNIRS) during a sustained-attention task in preschool children aged 3–6 years. By examining attentional performance and prefrontal HbO responses across age groups and motor-competence levels, the study tests whether behavioral improvements are accompanied by greater mean prefrontal activation and explores whether channel-level patterns are compatible with a neural-efficiency account.

In doing so, the present study advances current understanding in three key ways. First, it moves beyond descriptive embodied cognition accounts by specifying an association-based model of motor-attention coupling. Second, it challenges the prevailing assumption that better cognitive performance necessarily reflects increased metabolic demand, instead evaluating a neural-efficiency framework in early development. Third, it evaluates whether behavioral differences in attentional stability are accompanied by increased mean prefrontal HbO responses and explores whether the observed pattern is compatible with a neural-efficiency account.

## 2 Literature review

### 2.1 Embodied cognition and cognitive development

Embodied Cognition (EC) theory posits that cognitive processes are deeply embedded in the body's interactions with the environment. Unlike traditional views that treat cognition as abstract and separate from physical experience, EC emphasizes the integral role of bodily sensations, movements, and actions in shaping cognition (Meireles Santos da Costa et al., 2024). This perspective suggests that the mind is not solely confined to the brain but is distributed across the body and environment. It asserts that bodily movements, particularly sensorimotor activities, are directly linked to cognitive processes such as perception, attention, and memory (Zou et al., 2025).

Embodied cognition provides a framework for examining how motor competence and attentional regulation may be coupled within developing perception–action systems (Diamond, 2000, Gottwald et al., 2016). Attentional stability, which refers to the ability to maintain focus over extended periods, is crucial for various cognitive tasks and is closely related to motor development. The two systems—motor and cognitive—are tightly interconnected during early development (Song, 2019). While previous studies have explored the relationship between motor abilities and cognitive functions such as memory and executive functions (Zheng et al., 2023; Bao et al., 2024), the neurocognitive association that underlies the relationship between motor development and attentional stability remains largely underexplored. Addressing this gap is essential as it could provide deeper insights into how early motor experiences shape cognitive stability and performance.

Developmental neuroimaging studies have separately characterized neural systems supporting attentional control and motor function in children, but direct evidence linking individual differences in motor competence to attentional stability remains limited (Harrewijn et al., 2020, Urriola et al., 2025). fNIRS research indicates that motor-skill acquisition is accompanied by changes in cortical activation, but the extent to which such changes relate to attentional regulation remains unclear (Li et al., 2024). However, the specific neural association through which motor development influences attentional stability, particularly in young children, remains insufficiently understood. This gap provides an opportunity for further investigation into how embodied cognition might offer a framework for understanding the neurodevelopmental processes that connect motor skills with attentional control.

### 2.2 The relationship between motor development and attentional stability

Motor development plays a critical role in shaping cognitive functions, with attention being one of the most influential domains. For preschool-aged children, motor skills are closely linked to the ability to regulate and sustain attention (Pecuch et al., 2021). Early motor experiences, such as coordination, balance, and fine motor control, can significantly impact a child’s ability to focus, maintain attention, and resist distractions during cognitive tasks (Klupp et al., 2021; Ziereis and Jansen, 2015). Research consistently shows that children with better motor skills tend to perform better on attention tasks, particularly those that involve sustained attention and impulse control (Scharoun et al., 2013). This suggests that motor development not only predicts attention control but also contributes to the neural association underlying attentional stability.

Existing research indicates that the relationship between motor skills and attentional control has been associated with activity in brain regions involved in executive function, particularly the prefrontal cortex (PFC) (Ikkai and Curtis, 2011; Faw, 2003). The PFC is crucial for regulating attention, inhibiting irrelevant information, and maintaining focus over time. Neuroimaging research has shown that motor activities in early childhood are associated with activation in the PFC, which may underlie improvements in attention control as children develop (Kolb et al., 2012). However, while the link between motor development and attention has been established, the specific neural association—particularly how motor skills influence PFC activation—remains unclear. This gap in understanding presents an opportunity for further investigation, particularly in exploring how early motor experiences shape the neural substrates of attention regulation in children. Moriguchi and Hiraki (2013) reviewed developmental changes in prefrontal activation during executive-function tasks in young children, but this literature does not directly establish a motor–attention neural pathway. Embodied accounts nevertheless provide a testable rationale for examining whether motor competence and attentional performance are associated within developing perception–action systems (Gottwald et al., 2016).

In summary, although the association between motor development and attentional stability has been consistently documented, the neurodevelopmental association underlying this relationship-particularly the role of prefrontal cortical engagement across early childhood-remains insufficiently understood. Specifically, little is known about how individual differences in motor competence are reflected in prefrontal activation patterns during attentional processing, and whether such neural patterns change developmentally.

To address these gaps, the present study investigates the embodied link between motor development and attentional stability in preschool children by integrating behavioral assessments with functional near-infrared spectroscopy (fNIRS). The study addresses the following research questions:

(1) How is motor competence associated with task-based attentional stability in preschool-aged children, and are behavioral differences accompanied by differences in mean prefrontal HbO responses?
(2) Do age and motor competence interact in relation to attentional stability and mean prefrontal HbO responses during early childhood?

## 3 Methods

### 3.1 Participants

A total of 62 typically developing children (37 boys, 25 girls) aged between 3 and 6 years participated in this study. All participants were recruited from a public kindergarten in East China. Children were grouped into three age bands based on chronological age: early preschool (3.0–3.9 years), middle preschool (4.0–4.9 years), and late preschool (5.0–5.9 years). Descriptive statistics for age and gender distribution are summarized in Table 1. This study received ethical approval from the Ethics Committee of the School of Foreign Languages, China University of Petroleum (East China) (Approval No. UPCSFSEC2026033). Written informed consent was obtained from all primary caregivers prior to participation. All 62 participants provided complete behavioral data and were included in the behavioral analyses. One participant’s fNIRS dataset did not meet the predefined participant-level quality-control criteria and was therefore excluded from the fNIRS analyses only. Consequently, the behavioral analytic sample comprised 62 children, whereas the fNIRS analytic sample comprised 61 children.

**Table 1:** Participant demographics and age distribution by preschool stage.

Chronological Age	n	Mean Age (years) ± SD	Male	Female
Early Preschooler	17	3.24 ± 0.26	8	9
Middle Preschooler	27	4.03 ± 0.24	18	9
Late Preschooler	18	5.13 ± 0.42	11	7
Total	62	4.13 ± 0.78	37	25

To define sample eligibility and reduce major neurodevelopmental confounding, prespecified inclusion and exclusion criteria were applied. Inclusion criteria required that children: (1) were right-handed; (2) had normal or corrected-to-normal vision and hearing; (3) were born full-term (≥ 37 weeks of gestation); and (4) demonstrated the ability to understand and follow basic experimental instructions. Exclusion criteria included: (1) a reported history of neurological, psychiatric, or developmental disorders (e.g., ADHD or autism spectrum disorder), as confirmed by parental reports and teacher records; (2) current use of medications affecting the central nervous system; and (3) failure to meet the fNIRS quality-control criteria specified in Section 3.5.3. Criterion resulted in exclusion from the neural analyses only; valid behavioral data were retained in the behavioral analyses.

The study employed a developmental cross-sectional design with age group as a between-subject factor. Within each age group, children were descriptively classified into low, medium, and high motor-index levels using the age-group-specific mean ± 0.5 SD of the study-specific overall motor index. Because this stratification produced small and uneven age-by-motor cells, the categorical analyses were treated as exploratory and descriptive. Primary statistical inference treated the overall motor index as a continuous predictor and adjusted for chronological age.

### 3.2 Tasks and measures

#### 3.2.1 Motor competence assessment

Motor competence was assessed using the Peabody Developmental Motor Scales, Second Edition (PDMS−2) (Folio and Fewell, 2000), a standardized instrument suitable for children from birth to 71 months. The PDMS−2 comprises six subtests: Reflexes, Stationary, Locomotion, Object Manipulation, Grasping, and Visual-Motor Integration. Because the Reflexes subtest is designed for infants aged 0–11 months, it was not administered in the present sample. Accordingly, the present study used the five age-appropriate subtests: Stationary (30 items), Locomotion (89 items), and Object Manipulation (24 items), which constitute the Gross Motor domain, and Grasping (26 items) and Visual-Motor Integration (72 items), which constitute the Fine Motor domain.

For the present analyses, the raw score for each of the five administered subtests was converted to a percentage-of-maximum-possible (POMP) score using the following formula: subtest POMP score = raw subtest score / maximum possible raw subtest score × 100. This transformation placed the five subtests, which contained different numbers of items, on a common 0–100 scale. The gross motor index was calculated as the sum of the Stationary, Locomotion, and Object Manipulation POMP scores (range: 0–300), whereas the fine motor index was calculated as the sum of the Grasping and Visual-Motor Integration POMP scores (range: 0–200). The overall motor index was obtained by summing all five subtest POMP scores (range: 0–500). These indices were study-specific measures and should not be interpreted as age-normed PDMS-2 standard scores, age-equivalent scores, or the official Gross Motor Quotient, Fine Motor Quotient, or Total Motor Quotient.

#### 3.2.2 Cancellation task (Attentional Stability Test)

Task-based attentional stability was assessed using a paper-and-pencil cancellation task that required children to maintain attention while visually scanning the stimulus array and executing manual cancellation responses. The task consisted of a 25 × 25 matrix containing randomly distributed geometric shapes (circles, triangles, rectangles, squares, and trapezoids). During the material presentation, children were seated comfortably at a child-sized desk in a quiet testing room. The experimenter first provided a practice trial with a smaller 5 × 5 matrix to ensure the child fully understood the rules.

Once comprehension was confirmed, children were instructed to identify and cancel predefined target shapes as accurately and continuously as possible. Prior to the formal test, each child completed a brief practice session (approximately 30–60 s) using a simplified matrix to ensure full understanding of the task instructions. The experimenter provided standardized verbal instructions and corrective feedback during practice but not during the formal test phase. For the standard behavioral assessment, the task was performed continuously within a fixed duration of 3 min. The attentional-stability index used in behavioral analyses was calculated exclusively from the standard 3-min paper-and-pencil cancellation task. This behavioral assessment was distinguished from the shortened cancellation paradigm used during fNIRS recording. Children were instructed to proceed at their own pace without interruption, and no feedback was provided during task execution to avoid influencing attentional stability. Performance was quantified using an attentional-stability index (ASI), which integrated processing efficiency and response accuracy during the cancellation task. Processing efficiency was calculated as the number of scanned figures divided by the total task duration (180 s). Response accuracy was calculated as the number of correct cancellations minus errors, divided by the total number of target figures (125). The ASI was calculated as the product of these two components:

ASI=(Totalscannedfigures/180)*[(CorrectCancellations−Errors)/125]

All observed ASI values fell within the unit interval [0, 1], with four observations equal to 1. These boundary observations were adjusted before beta-regression analysis using the Smithson–Verkuilen transformation:

$${ASI}_{adjusted}=[ASI(n-1)+0.5]/n$$

where n represents the sample size (n = 62). The adjusted ASI values were used in subsequent beta regression analyses.

Thus, the attentional stability index should be interpreted as a task-based composite measure reflecting sustained task engagement under combined attentional, visual-scanning, processing-speed, and motor-response demands.

#### 3.2.3 fNIRS task paradigm

To accommodate neuroimaging constraints, the cancellation task was adapted into a block design while neural activity was simultaneously recorded using fNIRS. The experimental paradigm consisted of three cycles of alternating rest (20 s) and task (30 s) conditions. The fNIRS-adapted version was used only for neural activation analysis and was not used to calculate the behavioral attentional-stability index. Each task block involved continuous cancellation activity, during which children actively scanned the matrix and marked target shapes without interruption. No trial-by-trial segmentation was imposed, consistent with the sustained-attention design.

The presentation steps were carefully managed by the experimenter to minimize head movement. During rest periods, children were instructed to drop their pencils, remain still, and fixate on a centrally presented cross printed on a stand placed approximately 50 cm in front of them. During task blocks, the experimenter provided a standardized verbal cue (“Start finding the shapes!”) to initiate the task, and a second cue (“Stop and look at the cross!”) to terminate it. These cues were delivered in a consistent tone and timing across participants to ensure procedural standardization. No performance feedback was provided during the fNIRS recording session.

### 3.3 Data acquisition

Functional near-infrared spectroscopy (fNIRS) data were acquired using a NirSmart portable fNIRS system (Huichuang Medical, China). The system is equipped with dual-wavelength near-infrared light sources (730 nm and 850 nm) and a multichannel optode array, enabling continuous monitoring of cortical hemodynamic activity. Data were sampled at 11 Hz.

Participants were seated comfortably in a quiet, dimly lit room and were instructed to remain still and minimize head movements throughout the task. The optodes were mounted on a lightweight head cap and positioned according to the international 10–20 EEG system to ensure consistent channel placement across participants. The montage primarily covered prefrontal regions, including dorsolateral prefrontal and frontopolar areas, and also included adjacent channels approximately overlying premotor/motor-related and posterior cortical regions. Channel-to-region labels were used only as approximate scalp-projected anatomical descriptions based on template registration and were not used for confirmatory localization.

### 3.4 fNIRS data processing

fNIRS data preprocessing was conducted using the manufacturer-provided NirSmart analysis software, following established preprocessing procedures commonly adopted in pediatric fNIRS research. fNIRS signals were recorded continuously throughout the block-design paradigm. Each cycle consisted of a 20-s rest period followed by a 30-s task period. The onset of each task block, marked by the instruction to begin the cancellation task, was defined as 0 s.

The preprocessing pipeline included the following steps. First, non-task-related periods, including the initial adaptation phase and segments with excessive noise, were excluded. Second, motion-related artifacts were detected using a sliding-window approach. Specifically, abrupt changes in optical density exceeding 0.1 within a 1-s window were identified as motion artifacts and corrected using spline interpolation. Third, a band-pass filter of 0.01–0.20 Hz was applied. Fourth, optical density signals were converted into changes in oxygenated hemoglobin (HbO) and deoxygenated hemoglobin (HbR) using the modified Beer–Lambert law.

For each task block, baseline correction was performed by subtracting the mean signal during the 5 s immediately preceding task onset (−5–0 s) from the task-period signal. To quantify task-related neural activation, the primary dependent measure was defined as the mean baseline-corrected HbO response extracted from 2 to 32 s relative to task onset. This window captured the task-evoked hemodynamic response over the 30-s task period and the first 2 s after task offset, thereby accommodating the expected delay in the HbO response. Mean HbO values were first calculated for each valid task block and then averaged across valid blocks for each channel. These block-averaged mean HbO values were subsequently used in the channel-wise and ROI-based statistical analyses. For the primary analyses, HbO was retained because it is generally more sensitive to task-related cortical activation in pediatric fNIRS studies.

### 3.5 Statistical analysis

#### 3.5.1 Behavioral data analysis

Descriptive statistics were calculated for all behavioral variables, including gross motor, fine motor, and overall motor scores, and attentional stability indices. Prior to inferential analyses, data distributions were assessed for normality using the Shapiro-Wilk test and for homogeneity of variance using Levene’s test.

To examine developmental differences across age groups, one-way analyses of variance (ANOVA) were conducted for normally distributed variables with homogeneous variances. When the assumption of homogeneity of variance was violated, Welch’s ANOVA was applied. For variables that deviated from normality, appropriate non-parametric tests were used. Post-hoc comparisons were performed with correction for multiple comparisons to identify specific group differences.

Beta regression was used only for secondary and exploratory analyses of age-group differences and categorized motor-competence levels. Because several age-by-motor cells were small and uneven, these categorical analyses were not treated as the primary evidence for the motor–attention association. Boundary-adjusted ASI values were used in these beta-regression analyses.

Primary inference regarding the continuous association between motor competence and attentional stability was based on multiple linear regression using the full behavioral sample of 62 children. The untransformed attentional-stability index was entered as the dependent variable. The study-specific overall motor index and chronological age were z-standardized using the means and standard deviations of the full behavioral sample before model fitting. The model was specified as follows:

$${ASI}_{i}=\beta_{0}+\beta_{1}[z(overal\,l\;motor\;{index}_{i})]+\beta_{2}[z(chrono\,\log ical\;{age}_{i})]+\varepsilon_{i}.$$

As a sensitivity analysis, the same multiple linear regression model was repeated in the subset of 61 children whose fNIRS data passed the predefined quality-control criteria. This analysis was conducted to determine whether the behavioral association remained stable in the neural analytic sample.

To evaluate statistical sensitivity, a post hoc power (sensitivity) analysis was conducted using the pwr package in R. Given the sample size (N = 62) and α = .05, the analysis indicated that the study was adequately powered (1-β =.80) to detect moderate effect sizes in continuous associations (approximately r ≈ 0.30–0.35).

However, it is important to note that statistical power is substantially reduced in analyses involving categorical stratification into multiple subgroups. As such, subgroup comparisons based on the age × motor-level design should be interpreted cautiously, as small and uneven cell sizes may lead to unstable estimates and increased risk of Type II error.

#### 3.5.2 fNIRS data analysis

fNIRS data were analyzed using a region-of-interest (ROI)-based confirmatory approach, focusing on prefrontal regions that have been consistently implicated in attentional control and executive functioning. Channel-level activation mapping and group-level statistical comparisons were conducted using different HbO summary measures. For activation mapping, task-related HbO responses were estimated using a general linear model (GLM), and GLM-derived β values were used for within-group activation analyses. For ROI-level and channel-level group comparisons, participant-level mean baseline-corrected HbO responses averaged across the 2–32 s task window were used. An ROI-based strategy was adopted to reduce the risk of inflated Type I error associated with channel-wise multiple comparisons and to directly test theoretically associated hypotheses.

Based on prior developmental neuroimaging literature, two ROIs were defined: the dorsolateral prefrontal cortex (DLPFC) and the frontopolar cortex. Within each ROI, task-related activation was quantified as the mean baseline-corrected HbO response averaged across the predefined task-related window of 2–32 s after task onset. ROI-level mean HbO values were obtained by averaging the corresponding channels within each ROI. Because ROI-level HbO values were averaged at the participant level, each participant contributed one observation per ROI. Therefore, group-level analyses were conducted using general linear models rather than linear mixed-effects models. Separate linear models (GLMs) were fitted for the DLPFC and frontopolar ROI. The models included age group (early, middle, and late preschool), motor-competence level (low, medium, and high), their interaction, and gender as a covariate. The early-preschool group and low motor-competence level were specified as reference categories in the regression models.

Post-hoc comparisons were performed where appropriate. For exploratory channel-level analyses, Games–Howell post-hoc tests were conducted following significant Welch’s ANOVA results to account for unequal variances and unequal sample sizes among groups.

#### 3.5.3 Data quality control and exclusion criteria

All fNIRS data were subjected to rigorous quality assessment prior to statistical analysis. Channels and participants with excessive noise or insufficient valid signal across task blocks were excluded based on predefined quality thresholds. Specifically, an individual channel was excluded if its signal-to-noise ratio (SNR) fell below 3.0, or if cardiac pulsation was not visually detectable. For example, channels exhibiting flat-line signals or excessive high-frequency noise were considered invalid. At the participant level, a dataset was excluded if more than 30% of channels within predefined ROIs were invalid. Data quality control was conducted in a two-step procedure: first at the channel level, and subsequently at the participant level. Only participants with sufficient valid channels were retained for further statistical analyses. One of the 62 fNIRS datasets failed the predefined participant-level quality-control criteria and was excluded from all neural analyses. Accordingly, the fNIRS analytic sample comprised 61 children. The participant’s valid behavioral data were retained in the primary behavioral analysis.

## 4 Results

### 4.1 Motor competence, attentional stability, and prefrontal mean HbO responses

Categorical subgroup analyses are reported only to illustrate descriptive patterns. Because several age-by-motor cells were small and uneven, especially the low-motor-competence cell within the late-preschool group, these subgroup results were not used as the primary basis for inference. Primary inference was based on analyses treating motor competence as a continuous predictor while controlling for chronological age. Using the study-specific overall motor index derived from the five PDMS-2 subtest POMP scores, children within each age group were descriptively classified into low, medium, and high motor-competence levels based on the age-group-specific mean ± 0.5 SD. Specifically, for the early preschool group, scores were categorized as high (> 413.56), medium (386.21–413.56), and low (< 386.21). For the middle preschool group, the corresponding cutoffs were high (> 460.43), medium (433.14–460.43), and low (< 433.14). For the late preschool group, children were classified as high (> 496.26), medium (478.42–496.26), and low (< 478.42). This age-group-specific stratification reduced direct confounding by chronological age in the descriptive subgroup analyses but did not establish age-independent motor competence. Accordingly, primary inference was based on the continuous overall motor index with chronological age included as a covariate.

Descriptive statistics of attentional stability across motor-competence levels within each age group are presented in Table 2. In the early preschool group, children in the low motor-competence group showed substantially lower attentional stability (M = 0.457, SD = 0.163) than those in the medium (M = 0.642, SD = 0.188) and high motor-competence levels (M = 0.638, SD = 0.165), with the medium and high levels showing comparable means. A similar pattern emerged in the middle preschool group, where the low motor-competence level exhibited lower attentional stability (M = 0.579, SD = 0.175) relative to the medium (M = 0.661, SD = 0.148) and high levels (M = 0.656, SD = 0.179). Descriptively, the late preschool group appeared to show a graded pattern across motor-competence levels. However, this pattern should be interpreted cautiously because the low motor-competence cell was very small (n = 2), making the subgroup estimate unstable.

**Table 2:** Descriptive statistics of attentional stability by motor-competence level within each age group.

Ages	Motor Competence Levels	Attentional Stability		
		n	M	SD
Early Preschool	Low Level	7	0.457	0.163
	Medium Level	6	0.642	0.188
	High Level	4	0.638	0.165
Middle Preschool	Low Level	7	0.579	0.175
	Medium Level	11	0.661	0.148
	High Level	9	0.656	0.179
Late Preschool	Low Level	2	0.480	0
	Medium Level	11	0.700	0.161
	High Level	5	0.885	0.117

Note: Means and SDs are rounded to three decimals to preserve precision for small between-group differences.

**Fig. 1:**
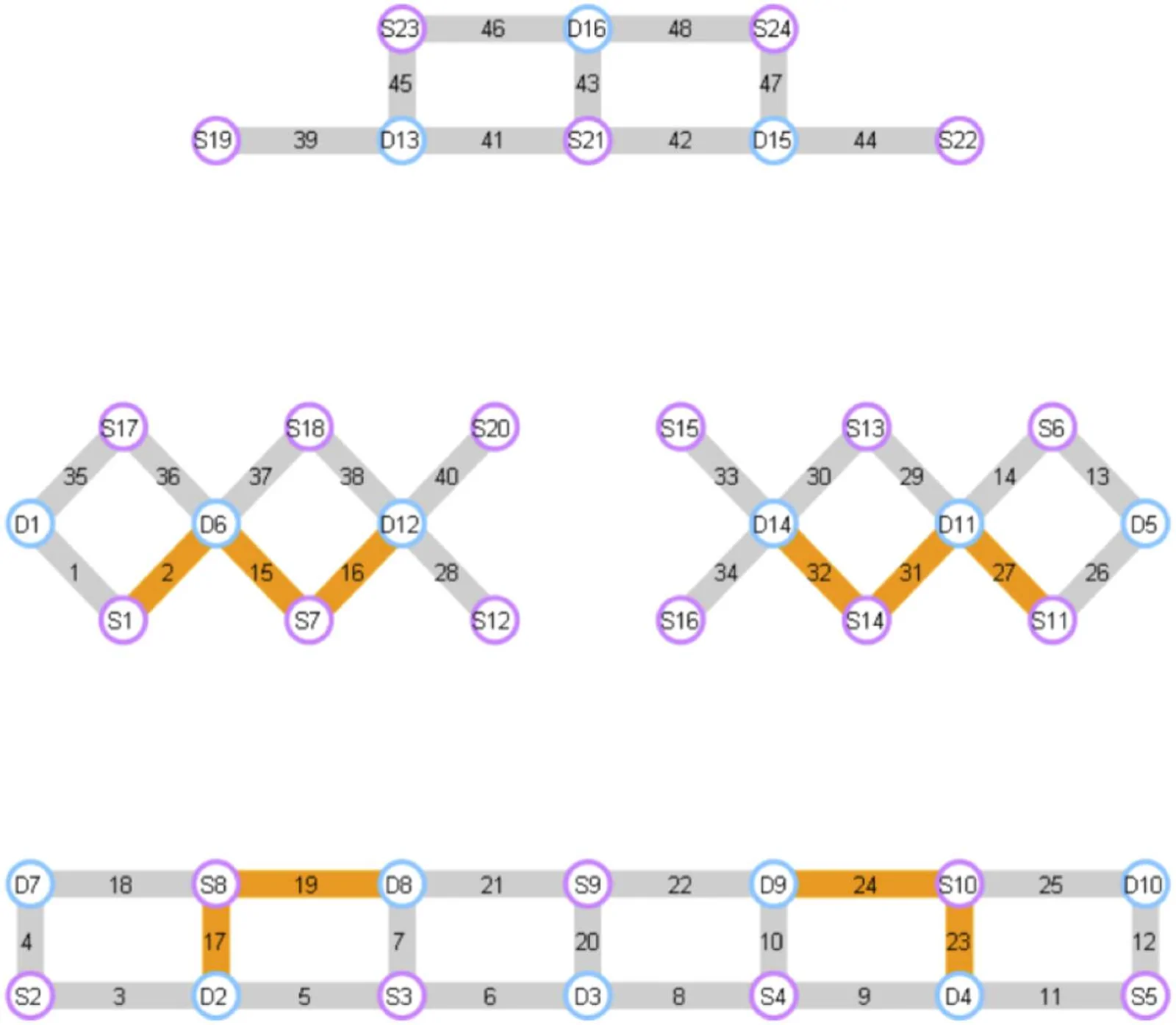
The distribution of DLPFC channels.

**Fig. 2:**
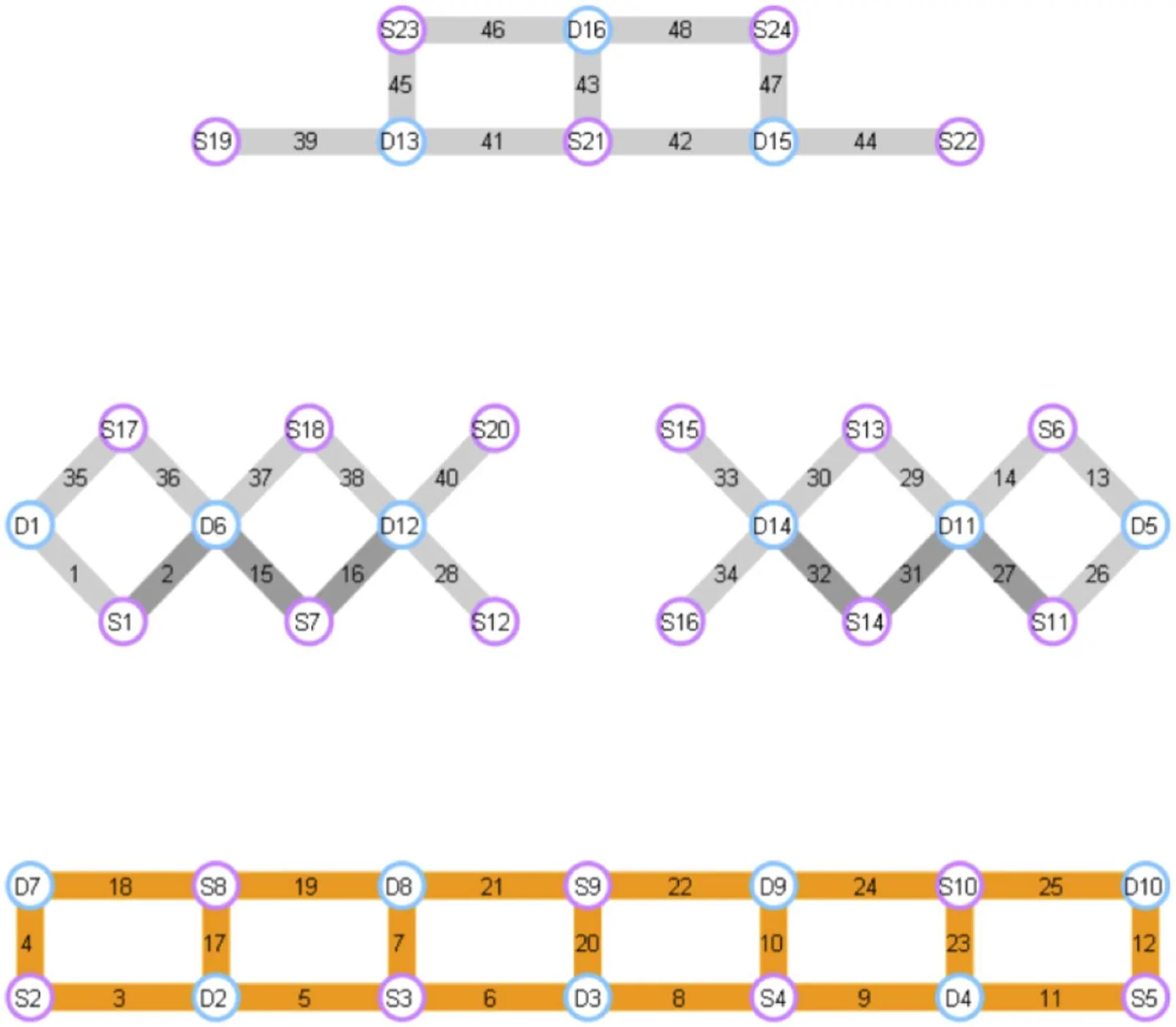
The distribution of Frontopolar channels.

**Fig. 3:**
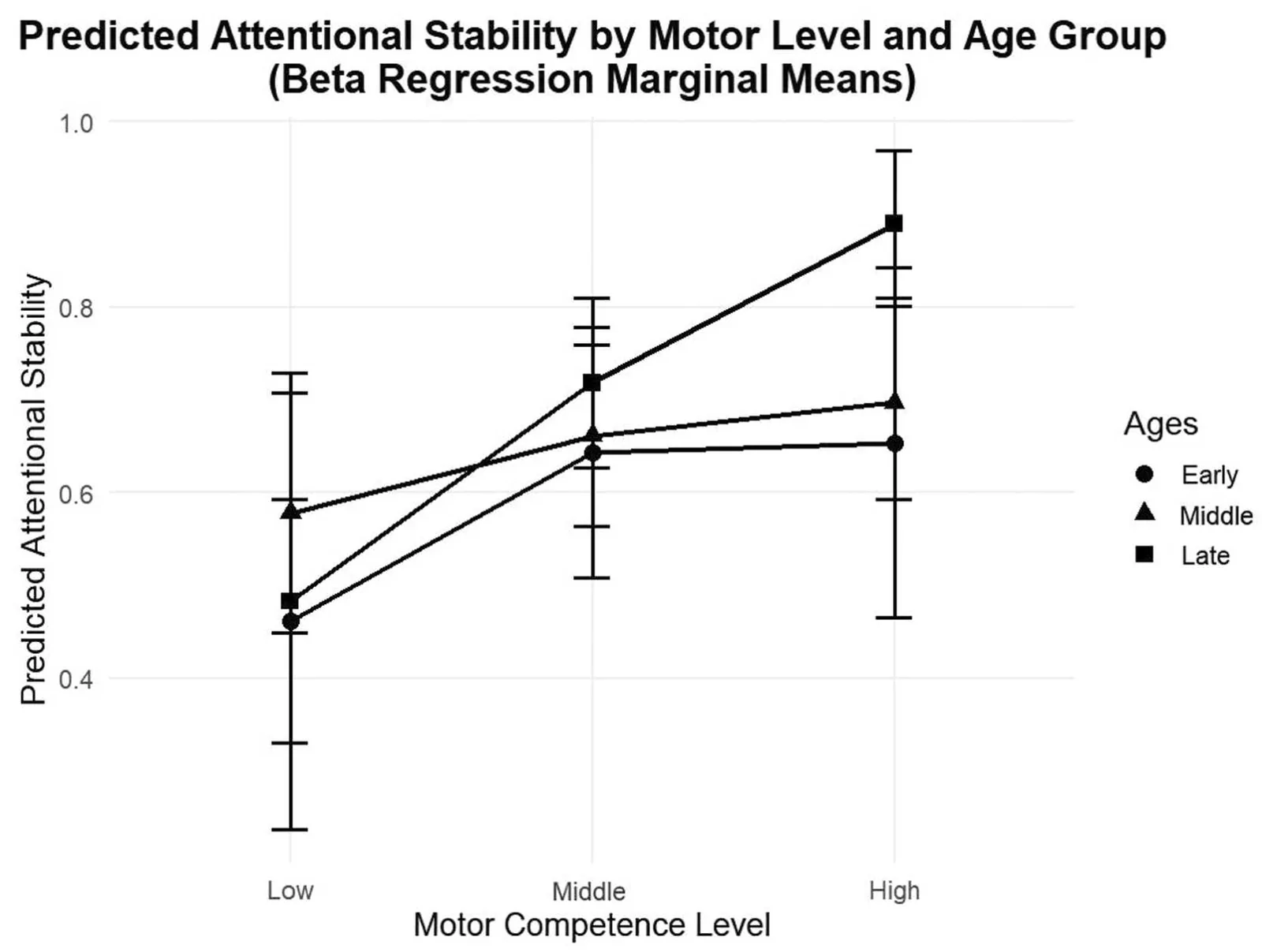
Exploratory model-estimated marginal means of attentional stability across categorized motor-competence levels by age group (beta regression, back-transformed).

Fig. 3 provides a descriptive visualization of the categorized motor-level pattern. Although predicted attentional stability tended to be higher at higher motor-competence levels, the descriptive separation appeared larger in the late-preschool group. However, the age group × motor-competence level interaction was not statistically significant, and this subgroup pattern should therefore be regarded as exploratory, particularly given the small and uneven cell sizes.

As a secondary and exploratory categorical analysis, a beta regression model with a logit link was fitted to examine age-group and categorized motor-competence effects. These results were not treated as the primary evidence for the continuous motor–attention association (Table 3A). Type II likelihood-ratio tests (Table 3B) showed a significant main effect of motor-competence level, χ²(2) = 11.92, *p* = .003. The main effect of age group was marginal, χ²(2) = 4.91, *p* = .086, and the age × motor-competence interaction was not significant, χ²(4) = 5.80, *p* = .214.

**Table 3A:** Exploratory beta-regression coefficients predicting attentional stability from categorized age and motor-competence levels.

Predictor	Estimate	SE	z	*p*
Intercept (Early × Low)	−0.153	0.269	−0.567	.571
Ages: Middle vs Early	0.468	0.382	1.225	.221
Ages: Late vs Early	0.085	0.570	0.149	.882
Motor Level: Medium vs Low	0.742	0.402	1.845	.065
Motor Level: High vs Low	0.788	0.503	1.567	.117
Ages Middle × Motor Medium	−0.389	0.533	−0.729	.466
Ages Late × Motor Medium	0.263	0.683	0.384	.701
Ages Middle × Motor High	−0.271	0.623	−0.434	.664
Ages Late × Motor High	1.367	0.820	1.666	.096
φ (precision)	6.826	1.178	5.797	< .001

Note: This categorized analysis was secondary and exploratory. Primary inference regarding the motor–attention association was based on the continuous multiple linear regression reported in the main text.

**Table 3B:** Type II likelihood-ratio tests for the exploratory categorical beta-regression model.

Effect	Df	χ²	*p*
Ages	2	4.910	.086
Motor Level	2	11.916	.003
Ages × Motor Level	4	5.801	.214

Because several age-by-motor cells were small and uneven, particularly the late-preschool low motor-competence cell, these categorical subgroup results should be interpreted cautiously and were not treated as the primary inferential evidence. Exploratory pairwise comparisons based on the categorized model suggested lower attentional stability in the low motor-competence group than in the medium and high motor-competence groups. We did not interpret simple effects within individual age groups as definitive evidence because the corresponding cell sizes were insufficient for stable subgroup inference.

To provide the primary continuous test of the motor–attention association, a multiple linear regression model was fitted using the full behavioral sample of 62 children. The untransformed attentional-stability index was entered as the dependent variable, while the z-standardized overall motor index and z-standardized chronological age were entered as predictors.

After controlling for age, the z-standardized overall motor index was a significant positive predictor of attentional stability (b = 0.083, SE = 0.031, t = 2.68, *p* = .010, 95% CI [0.021, 0.145]). Z-standardized chronological age was not a significant predictor (b = 0.005, SE = 0.031, t = 0.17, *p* = .863, 95% CI [−0.056, 0.067]). The model explained 22.4% of the variance in attentional stability (R² = .224, adjusted R² = .198), F(2, 59) = 8.53, *p* < .001. Because the overall motor index was z-standardized, its coefficient indicates that a one-standard-deviation increase in the motor index was associated with an estimated 0.083-unit increase in the original ASI scale. These findings indicate that the motor–attention association was not an artifact of subgroup categorization. As a sensitivity analysis, the regression was repeated in the subset of 61 children with usable fNIRS data. The untransformed ASI was retained as the dependent variable, while the overall motor index and chronological age were z-standardized before model fitting. The overall pattern remained unchanged: the z-standardized overall motor index remained a significant positive predictor of attentional stability after adjustment for age (b = 0.081, SE = 0.031, *p* = .011), whereas z-standardized chronological age was not significant (b = 0.005, SE = 0.031, *p* = .881). The model explained 21.3% of the variance in attentional stability (R² = .213, adjusted R² = .186), F(2, 58) = 7.87, *p* = .001.

To complement the ROI-based confirmatory analyses, exploratory channel-wise analyses were conducted to descriptively characterize task-related activation patterns across different levels of motor competence. These analyses were performed for visualization and pattern inspection purposes only and were not intended to support primary statistical inference.

Channel-level general linear model (GLM) analyses were conducted separately for children with low, medium, and high motor-competence levels. Within each group, one-sample *t*-tests were applied to GLM-derived HbO β values to identify channels exhibiting task-related activation, with false discovery rate (FDR) correction applied across channels (α=.05). The GLM modeled task blocks as regressors convolved with the canonical hemodynamic response function, with baseline terms included to account for signal drift.

**Fig. 4:**
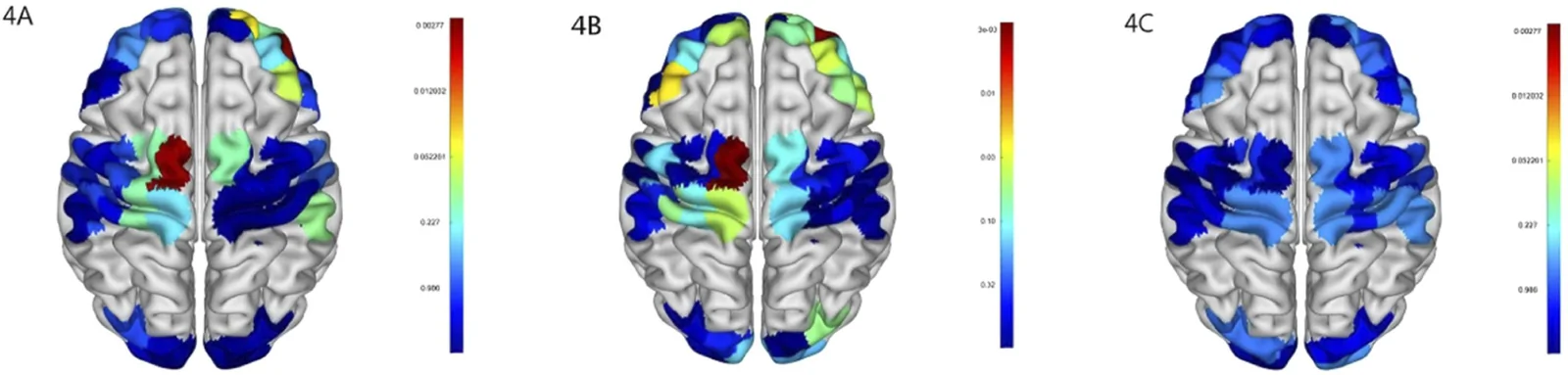
Task-related channel-wise activation maps by motor-competence level. Panels A–C show GLM-derived HbO β values for the low-, medium-, and high-motor-competence groups, respectively. Statistical significance was assessed using one-sample t tests with FDR correction across channels; significant channels are reported in Supplementary Tables S6A–S6C.

In the low motor-competence group, significant task-related activation was observed in a limited number of channels located over prefrontal and motor-related regions (e.g., frontopolar cortex, dorsolateral prefrontal cortex, pars triangularis, and premotor/supplementary motor areas; Fig. 4A, Supplementary Table S6A). The medium motor-competence group showed a broader spatial distribution of significant channels, including prefrontal, motor-related, and posterior regions (Fig. 4B, Supplementary Table S6B). In contrast, no channels survived FDR correction in the high motor-competence group (Fig. 4C), suggesting no suprathreshold channel-level activation under the present statistical threshold. This result should not be interpreted as direct evidence of reduced or more focal neural recruitment.

**Fig. 5:**
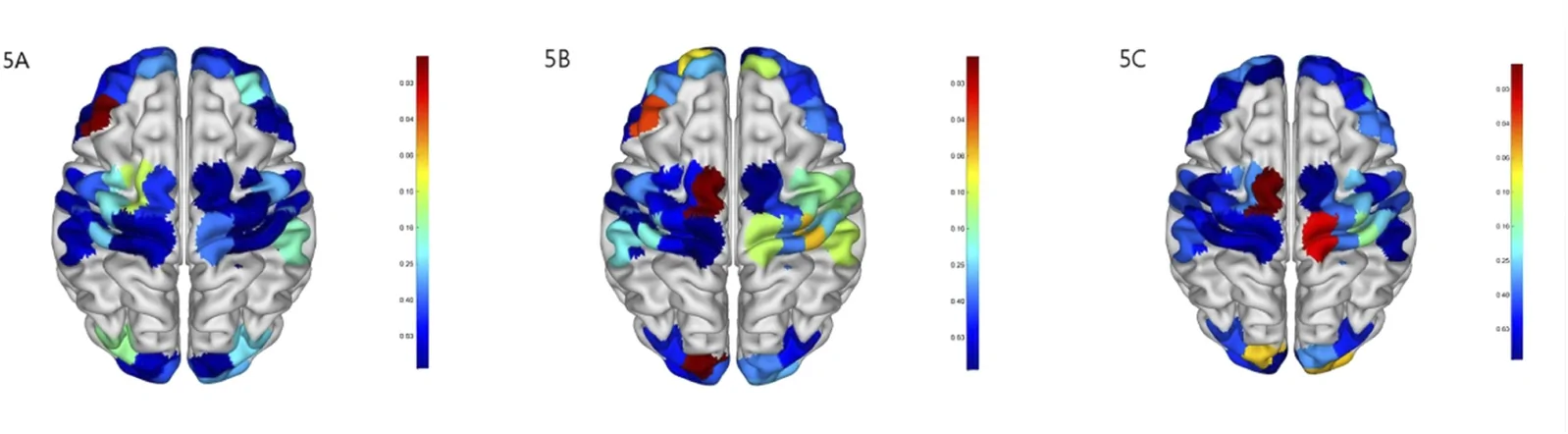
Exploratory pairwise differences in baseline-corrected HbO responses across motor-competence groups. Panels A–C show low versus medium, medium versus high, and low versus high comparisons, respectively. Welch’s ANOVA and Games–Howell post hoc tests were used. Because no multiplicity correction was applied across the 48 channel-wise omnibus tests, the maps are descriptive and should not be interpreted as confirmatory evidence of group differences.

For descriptive between-group comparisons at the channel level, assumption checks revealed heterogeneity of variance in a subset of channels (see Supplementary Tables S3-S4). Accordingly, Welch’s ANOVA was applied to examine group effects across channels. Because channel-wise analyses involved multiple spatial comparisons and were conducted for exploratory purposes, these findings were interpreted as descriptive spatial patterns rather than confirmatory effects. Nominal group differences were observed in selected channels in pairwise comparisons (low vs. medium, medium vs. high, and low vs. high motor-competence levels; Fig. 5, Supplementary Tables S7A-S7C). The topographic maps in Fig. 5 represent the spatial distribution of between-group HbO differences rather than absolute activation levels. Given the exploratory nature of these analyses and the large number of channel-level tests, these effects should be interpreted cautiously and are reported to illustrate potential spatial trends rather than definitive group differences. Taken together, the channel-wise analyses revealed descriptive differences in suprathreshold channel-level activation across motor-competence levels. These exploratory findings complement the ROI-based analyses but should not be interpreted as definitive evidence of spatial refinement or neural efficiency.

### 4.2 Age-related differences in behavioral performance and prefrontal mean HbO responses

Behavioral analyses in this section included all 62 children, whereas the fNIRS analyses included the 61 children whose datasets passed the predefined quality-control criteria.

**Fig. 6:**
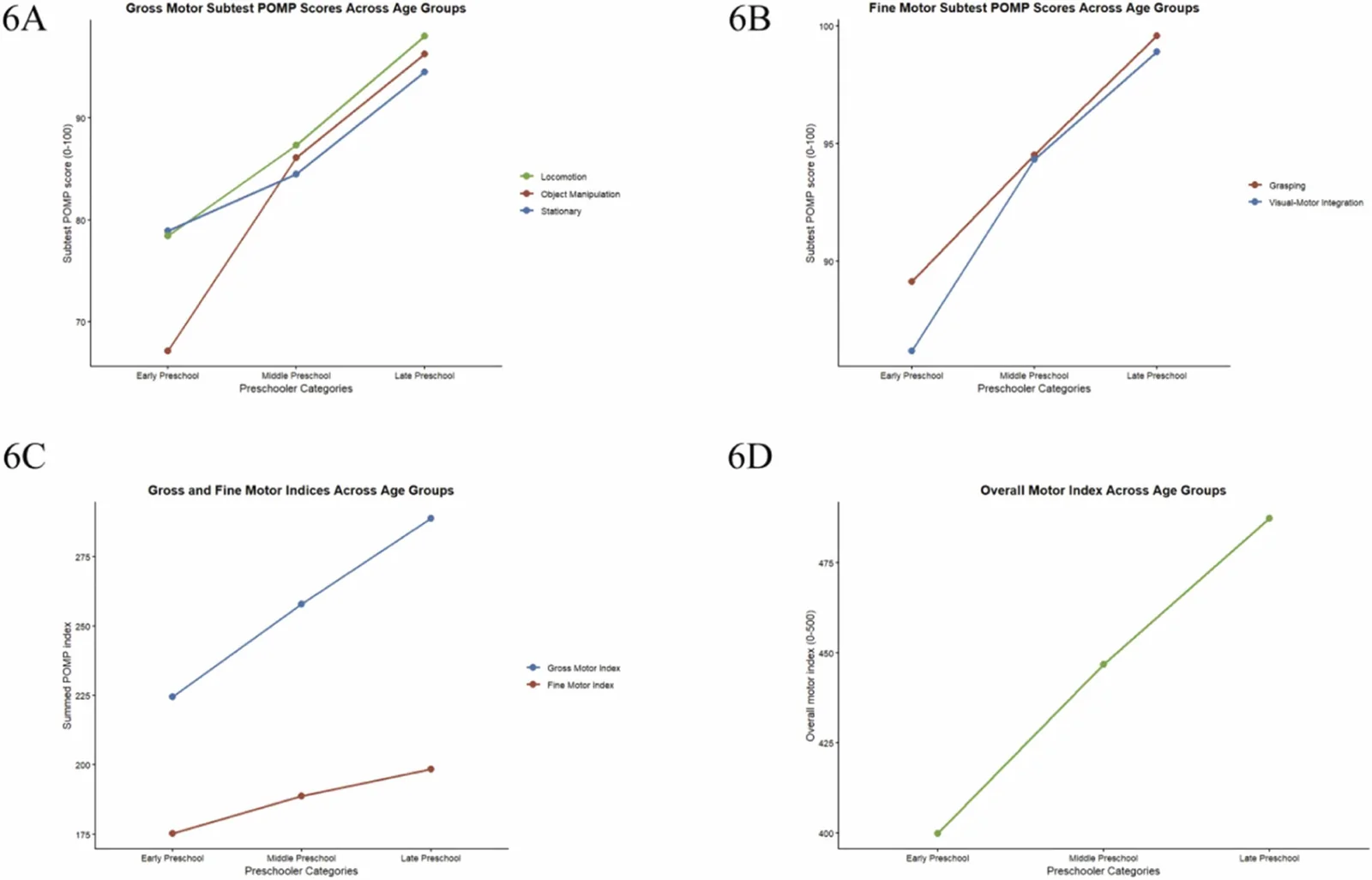
Age-related differences in study-specific PDMS-2-derived percentage-of-maximum-possible (POMP) scores and summed motor indices across preschool age groups. (A) Stationary, Locomotion, and Object Manipulation POMP scores; (B) Grasping and Visual-Motor Integration POMP scores; (C) gross motor index (range: 0-300) and fine motor index (range: 0-200); and (D) overall motor index (range: 0-500).

As illustrated in Fig. 6, the study-specific subtest POMP scores and summed motor indices increased across age groups. For the gross motor subtests, the late-preschool group showed the highest Stationary, Locomotion, and Object Manipulation scores, followed by the middle- and early-preschool groups. A similar pattern was observed for Grasping and Visual-Motor Integration. The gross motor index, fine motor index, and overall motor index also increased progressively from early to late preschool. Because these study-specific indices were not age-normed, they should be interpreted as measures of observed motor performance rather than as PDMS-2 norm-referenced standard scores or quotient scores.

Levene’s test indicated a violation of the homogeneity of variance assumption for overall motor scores (*p* = .043). Therefore, robust Welch’s one-way ANOVA was conducted, showing a significant effect of age group on overall motor development, F(2, 36.15) = 63.83, *p* < .001 (Brown–Forsythe test yielded a consistent result, F(2, 50.11) = 56.30, *p* < .001). For reference, the conventional one-way ANOVA also indicated a large age effect, F(2, 59) = 53.75, *p* < .001, η² = .65. Games–Howell post-hoc comparisons further showed that the middle preschool group scored significantly higher than the early preschool group, and the late preschool group scored significantly higher than both the early and middle preschool groups (all *ps* < .001; Table 4).

**Table 4:** Welch’s one-way ANOVA results for the study-specific overall motor index, calculated as the sum of five subtest POMP scores, across age groups.

	Early Preschooler (N = 17)		Middle Preschooler (N = 27)		Late Preschooler (N = 18)		ANOVA Results	Post Hoc (Games-Howell)
Overall motor index (range: 0–500)	M	SD	M	SD	M	SD	F(2,36.15) = 63.83***	Middle Preschooler > Early Preschooler Late Preschooler>Middle Preschooler Late Preschooler > Early Preschooler
	399.88	27.35	446.78	27.29	487.34	17.84		

Note: The overall motor index was calculated by summing the five subtest percentage-of-maximum-possible scores. It is a study-specific index rather than the official PDMS-2 Total Motor Quotient.

*** *p* < .001

Taken together, these results demonstrate robust age-related differences in observed gross- and fine-motor performance between 3 and 6 years of age, establishing a clear developmental gradient that provides a necessary behavioral foundation for subsequent analyses linking motor competence to attentional stability and neural activation patterns.

**Fig. 7:**
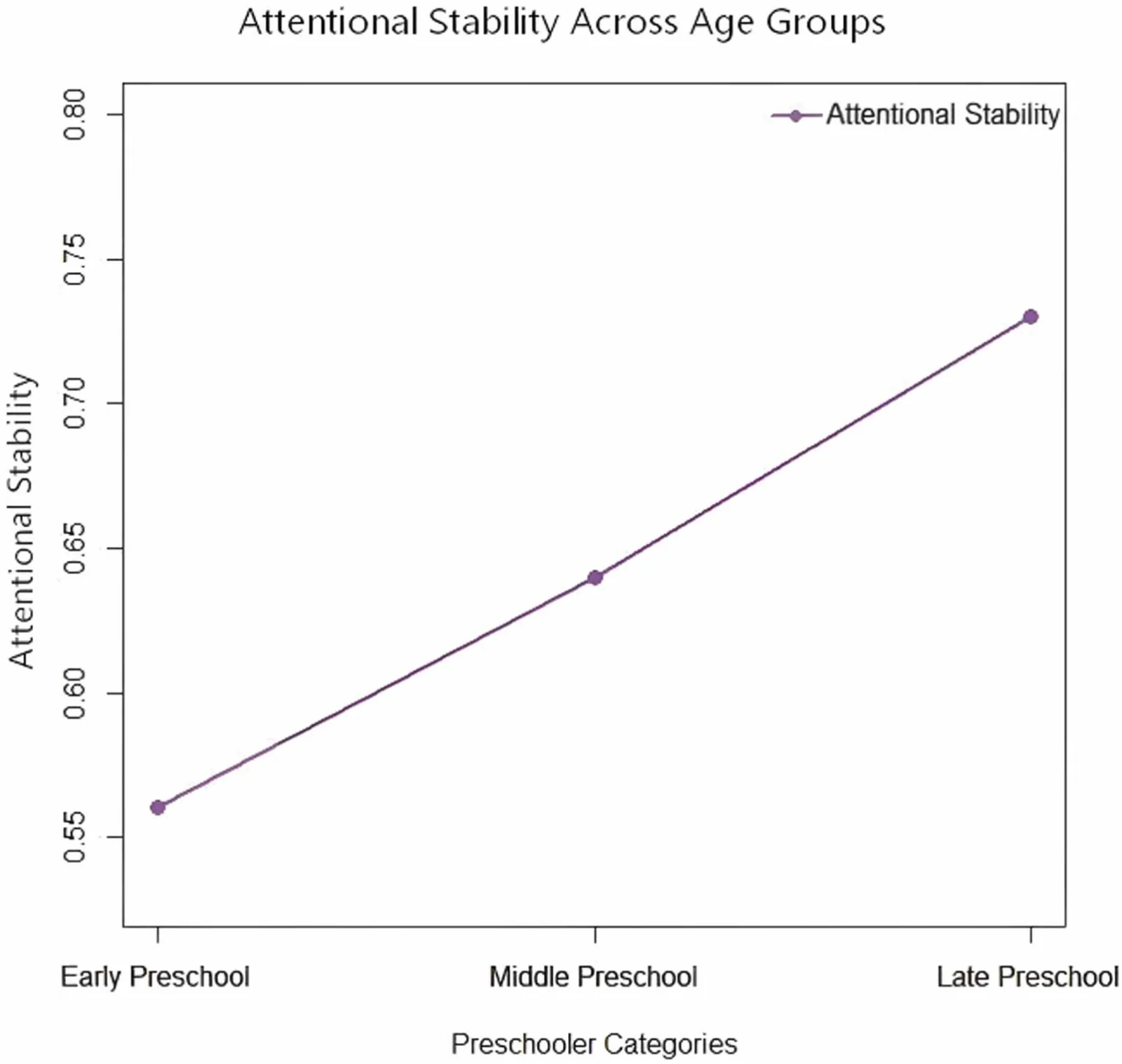
Observed attentional stability across age groups.

To examine developmental differences in attentional stability across preschool age groups, an attentional-stability index was computed based on the cancellation task (see Section 3.2.2 for details of the calculation). Descriptively, children in the late preschool period exhibited higher attentional stability scores than those in the early and middle preschool groups (Fig. 7).

A beta regression model was fitted to examine age-related differences in attentional stability. The overall effect of age was statistically significant, χ²(2) = 9.88, *p* = .007, indicating overall differences among age groups in attentional stability. Pairwise comparisons revealed that late preschoolers showed significantly higher attentional stability than early preschoolers (β= 0.88, SE = 0.28, z = 3.13, *p* = .0018). In contrast, the difference between middle and early preschoolers was not statistically significant (β= 0.35, SE = 0.25, *p* = .163), nor was the difference between late and middle preschoolers (*p* = .219). The estimated precision parameter was φ = 5.26 (SE = 0.88) (Table 5).

**Table 5:** Secondary beta-regression coefficients predicting attentional stability from age group.

Predictor	Estimate	SE	z	*p*
Intercept (Early)	0.258	0.193	1.34	.181
Middle vs Early	0.347	0.249	1.39	.163
Late vs Early	0.881	0.282	3.13	.0018
φ (precision)	5.259	0.883	5.96	< .001

**Fig. 8:**
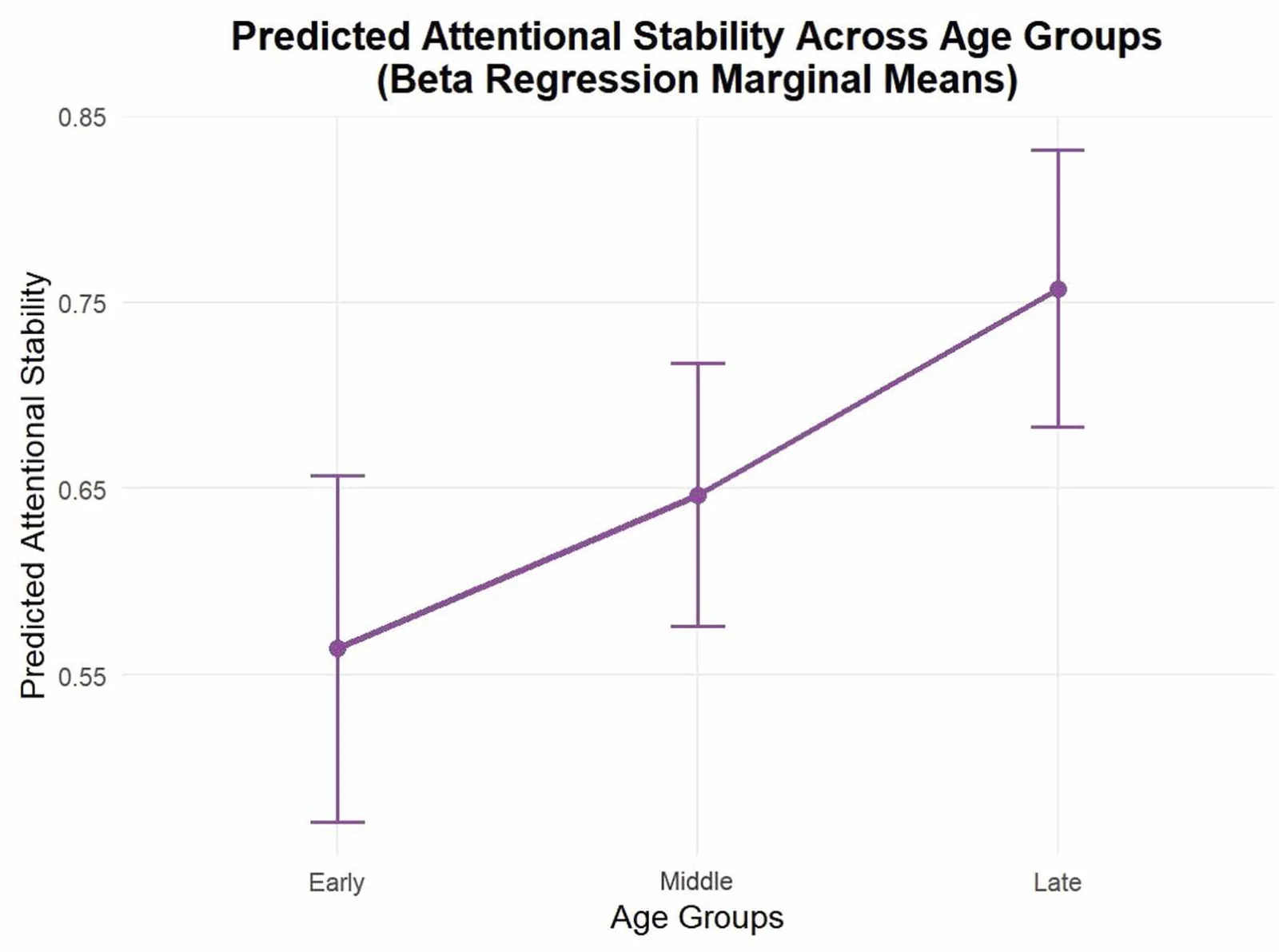
Model-estimated marginal means of attentional stability by age group from the secondary beta-regression analysis.

Model-estimated marginal means of attentional stability, back-transformed to the original 0–1 scale, are presented in Fig. 8. The estimated means demonstrate a gradual increase in attentional stability from early to late preschool, with a clear separation between the early and late groups. In contrast, substantial overlap in confidence intervals was observed between the early and middle preschool groups, suggesting a more pronounced developmental shift in attentional stability toward the later preschool period.

To provide a descriptive overview of channel-level activation patterns, exploratory channel-wise analyses were conducted across individual channels. These analyses are reported for completeness and visualization and pattern inspection purposes only and were not used as the primary basis for statistical inference.

**Fig. 9:**
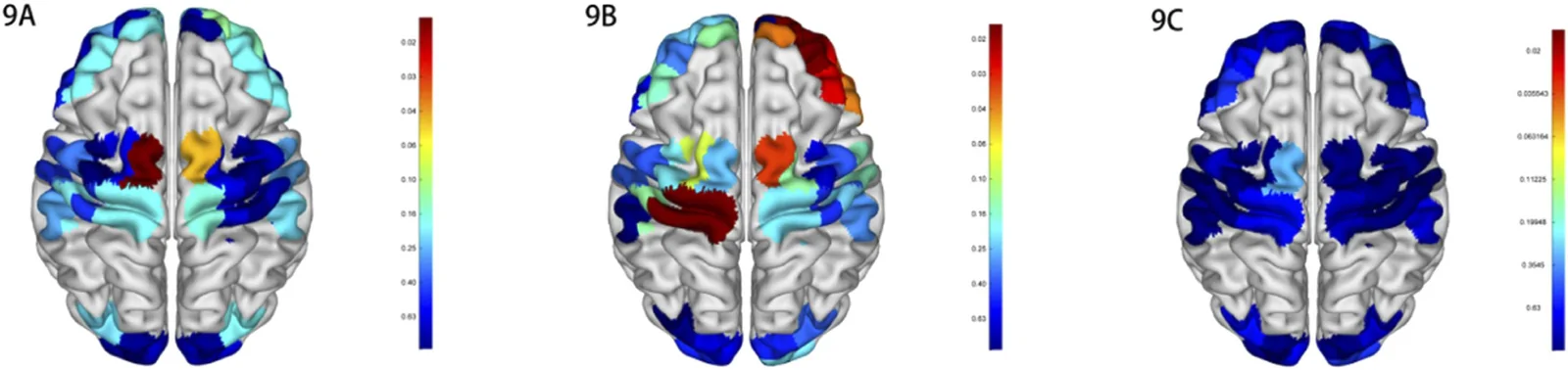
Task-related channel-wise activation maps by age group. Panels A–C show GLM-derived HbO β values for the early-, middle-, and late-preschool groups, respectively. Statistical significance was assessed using one-sample t tests with FDR correction across channels; significant channels are reported in Supplementary Tables S5A–S5C.

Within each age group (early, middle, and late preschool), task-related HbO responses were first estimated at the channel level using a general linear model (GLM). One-sample *t*-tests were then applied to the GLM-derived β values to identify channels showing task-related activation, with false discovery rate (FDR) correction applied across channels within each group (α =.05). In the early preschool group, significant task-related activation was observed in a limited set of channels primarily located over premotor and motor-related regions (e.g., channels S12–D12 and S16–D14; Fig. 9A, Supplementary Table S5A). In the middle preschool group, a broader pattern of channel-level activation emerged, encompassing channels over prefrontal and motor-related areas, including dorsolateral prefrontal and frontopolar regions (Fig. 9B, Supplementary Table S5B). In contrast, no channels survived FDR correction in the late preschool group (Fig. 9C), indicating that no channel-level activation reached the FDR-corrected threshold in this group. This finding should be interpreted cautiously and does not by itself demonstrate a more spatially restricted activation pattern.

To further describe potential between-group differences at the channel level, assumption checks (Shapiro–Wilk tests for normality and Levene’s tests for homogeneity of variance) were conducted for each channel (see Supplementary Tables S1 and S2). Given evidence of heteroscedasticity in several channels, Welch’s ANOVA was applied to test age-group effects across all 48 channels. Only channel S8–D2 showed a nominal omnibus effect of age group (Welch’s F(2, 34.60) = 4.054, *p* = .026); however, subsequent Games–Howell post hoc comparisons did not reveal significant pairwise differences between age groups (all *p*s > .05; Table 6).

**Table 6:** Welch’s one-way ANOVA results for Channel S8-D2 across age groups.

	F value	df (1)	df (2)	*p* value	Effect Size (η²)	Post Hoc (Games-Howell)
Channel S8-D2	4.054	2	34.60	.026	.190	No significant pairwise differences

Taken together, the channel-wise analyses revealed limited and inconsistent age-related differences at the individual-channel level. Given the exploratory nature of these analyses and the absence of robust post hoc effects, these findings should be interpreted with caution and are intended primarily to complement the ROI-based linear model results.

At the ROI level, linear models were fitted to examine the effects of age group and motor-competence level on task-related HbO responses in the DLPFC and frontopolar cortex. Age group, motor-competence level, and their interaction were entered as predictors, with gender included as a covariate.

#### 4.2.1 Dorsolateral prefrontal cortex (DLPFC)

**Fig. 10:**
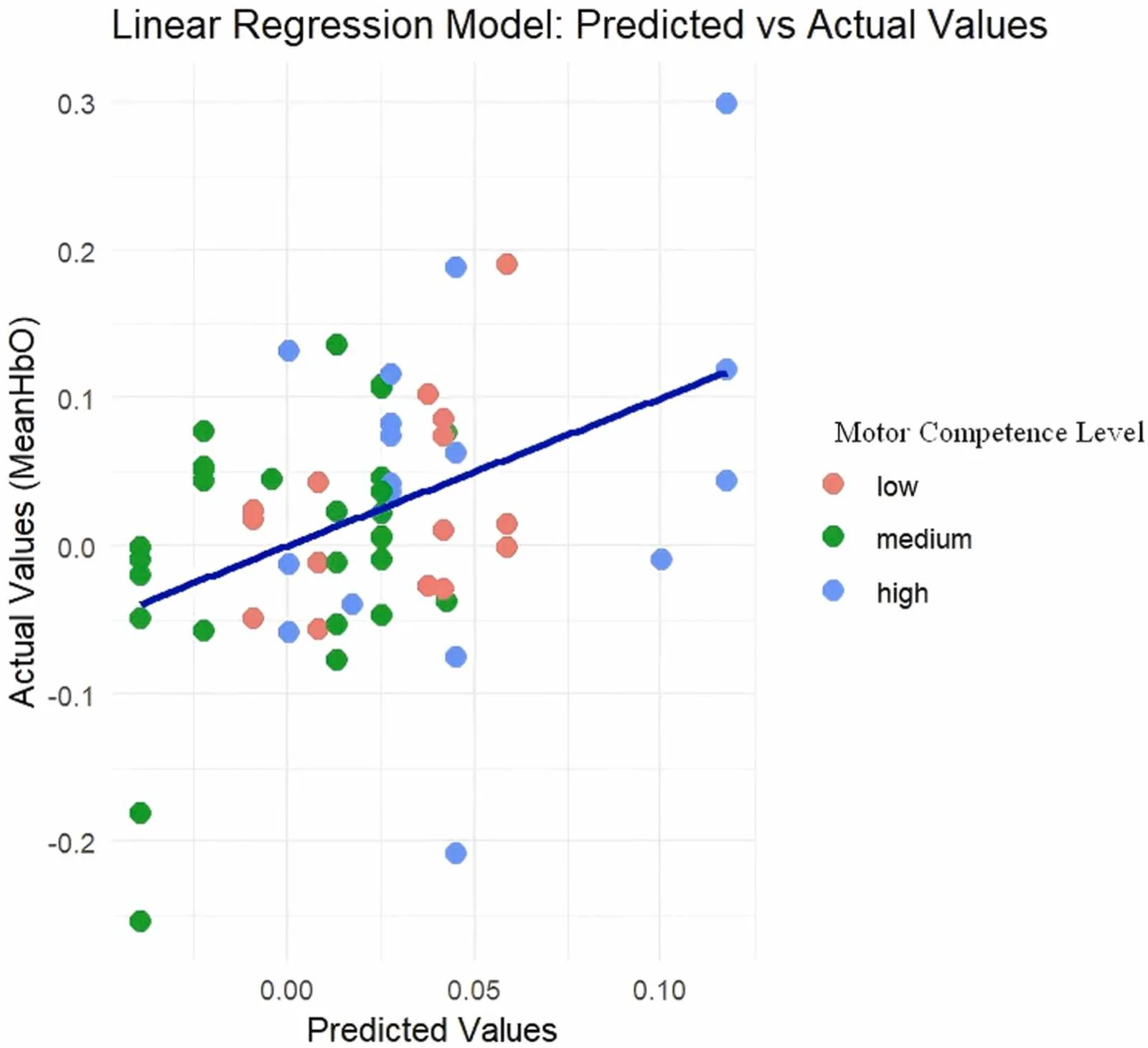
DLPFC mean HbO model: Predicted values and actual values in Linear Regression Model.

In the DLPFC model, no individual regression coefficient for age group, motor-competence level, or their interaction reached statistical significance. Gender was also not a significant predictor of mean DLPFC HbO responses (all *ps* >.05; Table 7, Fig. 10).

**Table 7:** Linear model results for mean HbO responses in the DLPFC among participants with usable fNIRS data (N = 61).

Predictor	Estimate (β)	Standard Error (SE)	t Value	*p* Value
Intercept	−0.008826	0.034857	−0.253	0.801
Age: Middle	0.050727	0.046913	1.081	0.285
Age: Late	0.046652	0.071179	0.655	0.515
Motor: Medium	0.005024	0.049864	0.101	0.920
Motor: High	0.009431	0.055191	0.171	0.865
Gender (Female = 1)	0.017094	0.024977	0.684	0.497
Age: Middle × Motor: Medium	−0.021452	0.066706	−0.322	0.749
Age: Late × Motor: Medium	−0.082060	0.083292	−0.985	0.329
Age: Middle × Motor: High	−0.023287	0.070753	−0.329	0.743
Age: Late × Motor: High	0.053133	0.096650	0.550	0.585

#### 4.2.2 Frontopolar cortex

**Fig. 11:**
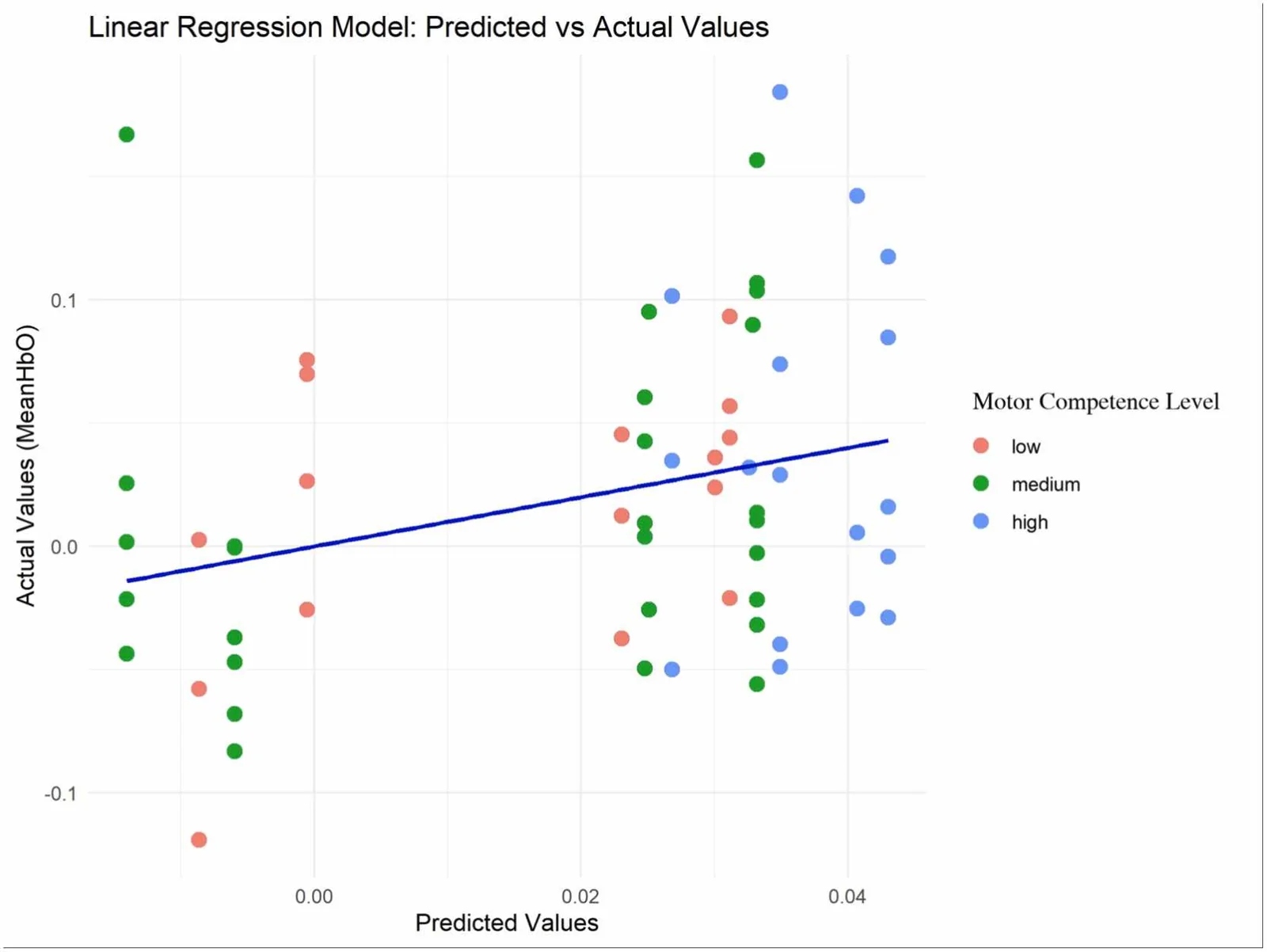
Frontopolar mean HbO model: Predicted values and actual values in Linear Regression Model.

In the frontopolar model, no individual regression coefficient for age group, motor-competence level, or their interaction reached statistical significance. Gender was also not significantly associated with mean frontopolar HbO responses. (all *ps* >.05; Table 8, Fig. 11).

**Table 8:** Linear model results for mean HbO responses in the frontopolar cortex among participants with usable fNIRS data (N = 61).

Predictor	Estimate (β)	Standard Error (SE)	t Value	*p* Value
Intercept	−0.000539	0.0265	−0.0203	0.984
Age: Middle	0.0317	0.0357	0.887	0.379
Age: Late	0.0306	0.0542	0.564	0.575
Motor: Medium	0.0334	0.0380	0.880	0.383
Motor: High	0.0412	0.0420	0.981	0.331
Gender (Female = 1)	−0.00809	0.0190	−0.426	0.672
Age: Middle × Motor: Medium	−0.0314	0.0508	−0.618	0.539
Age: Late × Motor: Medium	−0.0694	0.0634	−1.09	0.279
Age: Middle × Motor: High	−0.0294	0.0539	−0.545	0.588
Age: Late × Motor: High	−0.0364	0.0736	−0.494	0.623

The model did not detect statistically significant coefficient differences in mean frontopolar HbO responses across the reported age-group and motor-competence contrasts.

### 4.3 Integrated summary of behavioral and fNIRS findings

Behavioral analyses showed that higher continuous motor competence was associated with greater task-based attentional stability after controlling for chronological age. The descriptive separation across categorized motor-competence levels appeared larger in the late-preschool group, but the age × motor-competence interaction was not statistically significant.

At the neural level, none of the reported regression coefficients for age group, motor-competence level, or their interaction reached statistical significance in either ROI. Exploratory channel-wise analyses revealed different suprathreshold activation patterns across age and motor-competence groups; however, these analyses were descriptive and were not used as the primary basis for statistical inference.

## 5 Discussion

The present study addressed two questions concerning the association between motor competence, attentional stability, and prefrontal activation during early childhood. Regarding the first question, higher motor competence was associated with greater task-based attentional stability after chronological age was controlled. Although the descriptive separation across motor-competence levels appeared larger among late preschoolers, the age × motor-competence interaction was not statistically significant. The findings therefore support an overall motor–attention association but do not show that this association becomes stronger with age. This pattern is consistent with previous evidence linking motor competence to executive and attentional functioning in preschool children.

It is important to clarify the measurement scope of the present study. The attentional stability index derived from the cancellation task should be interpreted as a composite behavioral measure rather than a process-pure index of sustained attention. Task performance inherently involves multiple components, including visual scanning, processing speed, and motor execution (Deodhar and Bertenthal, 2023). As such, attentional stability in the present study reflects task-based performance under combined cognitive and motor demands, rather than a purely attentional construct.

### 5.1 Motor development and attentional stability: behavioral evidence within an embodied framework

Consistent with previous developmental research, both motor ability and attentional stability exhibited systematic age-related differences, with children in the late preschool period demonstrating more advanced motor skills and greater attentional stability than younger children (Diamond, 2000; Houwen et al., 2017; Oberer et al., 2017). Beyond these age-group differences, the continuous overall motor index was positively associated with task-based attentional stability after adjustment for chronological age.

From an embodied cognition perspective, this behavioral coupling suggests that motor development and attentional regulation may be supported by partially overlapping sensorimotor and executive processes (Piek et al., 2004; Cameron et al., 2012). Through repeated action-perception cycles, children with more advanced motor skills may develop more efficient coordination between bodily actions and environmental demands, thereby facilitating sustained attentional engagement. Rather than reflecting independent developmental trajectories, motor and attentional systems may co-develop in a mutually reinforcing manner, and improvements in motor control may provide a behavioral foundation for more stable and goal-directed attention.

Notably, the descriptive subgroup pattern showed a larger separation in attentional stability across motor-competence levels in the late-preschool group. However, the age group × motor-competence level interaction was not statistically significant, and the late-preschool low motor-competence cell was very small (n = 2). The apparent late-preschool pattern should therefore be interpreted as exploratory rather than as evidence that the motor–attention association strengthens with age.

This is particularly relevant given that the continuous overall motor index served as the primary predictor in the present study. Because the cancellation task required both visual scanning and manual cancellation responses, the observed association between motor competence and attentional stability may partly reflect shared task demands. Therefore, the motor–attention relationship should be interpreted as an association between motor competence and task-based attentional stability, rather than as evidence that motor competence predicts a process-pure measure of sustained attention.

### 5.2 Interpretation of prefrontal activation findings and alternative explanations

The ROI-based linear models did not identify any statistically significant individual coefficient for age group, motor-competence level, or their interaction in the DLPFC or frontopolar cortex. These null coefficient tests do not establish neurophysiological equivalence across groups and may reflect limited statistical power or measurement sensitivity.

Collectively, the behavioral model identified a motor–attention association, whereas the ROI models did not detect corresponding coefficient differences in mean prefrontal HbO responses. Importantly, these HbO responses should be more cautiously interpreted as reflecting task-related prefrontal engagement under sustained performance demands, rather than as a direct index of attentional control. Given the limited spatial resolution of fNIRS and the focus on superficial cortical regions, it is not possible to isolate specific cognitive processes or to capture broader network-level dynamics (e.g., frontoparietal or cerebellar systems). In addition, the reliance on HbO signals alone, without complementary HbR measures or functional connectivity analyses, further constrains neurophysiological interpretation and limits mechanistic inference.

The exploratory channel-wise analyses are presented for descriptive purposes and provide complementary information regarding spatial activation patterns. Younger children showed several suprathreshold channels, whereas no channels survived FDR correction in older children under the present threshold. This pattern should be interpreted as a descriptive channel-level result rather than definitive evidence of more spatially restricted activation. However, these patterns should be interpreted cautiously and not as definitive evidence of group-level neural differences.

Taken together, these findings may be broadly consistent with a neural efficiency account; however, this interpretation remains provisional and indirect. Alternative explanations should also be considered. The absence of detected coefficient differences may reflect relatively invariant task demands, limited measurement sensitivity, or ceiling or floor effects in developmental hemodynamic responses.

Importantly, the present study does not directly test the relationship between behavioral performance and neural activation at the individual level. Without explicit performance–activation coupling metrics, it is not possible to determine whether improved behavioral outcomes are associated with more efficient neural processing. As such, neural efficiency should be considered as one of several plausible interpretations of the observed pattern, rather than a definitive explanation.

From this perspective, one possible interpretation is that age and motor competence may be related to differences in how task-related neural resources are organized. However, the present channel-wise results do not provide direct evidence for spatial selectivity, and this interpretation remains provisional (Du et al., 2025; Han et al., 2022). Such differences may reflect variation in how neural resources are organized during task engagement, rather than differences in overall activation magnitude.

Because the study was cross-sectional and did not directly model individual-level performance–activation coupling, the neural findings should not be interpreted as evidence of within-child developmental change or network-level reorganization.

### 5.3 Embodied-cognition implications of the motor–attention association

The ROI models did not identify any statistically significant individual coefficient for age group, motor-competence level, or their interaction in either region. This null coefficient pattern does not establish neurophysiological equivalence across groups and provides only indirect evidence relevant to a neural-efficiency account (Yu and Liu, 2026). However, the absence of statistically significant effects should not be interpreted as evidence of neurophysiological equivalence across groups. Limited statistical power, relatively invariant task demands, developmental variability in task-related prefrontal recruitment, and restricted measurement sensitivity may all have contributed to the observed pattern (Moriguchi and Hiraki, 2013).

### 5.4 Limitations and future directions

First and foremost, the cross-sectional nature of the present design inherently limits the ability to draw developmental or causal inferences. The observed age-related differences reflect between-group variation and do not permit conclusions about within-child developmental trajectories, temporal ordering, or neural change over time. As such, interpretations related to developmental processes, including potential shifts in neural organization or efficiency, should be understood as theoretically informed but not directly demonstrated. Future longitudinal and intervention-based designs will be essential to establish whether improvements in motor competence are associated with within-subject changes in neural activation patterns. In particular, subgroup estimates derived from highly stratified designs should be interpreted with caution, as small cell sizes may produce unstable parameter estimates and limit reproducibility. Furthermore, other factors such as individual differences in processing speed, motivation, and fatigue may have influenced task performance and were not independently controlled in the present design. These factors may contribute additional variability to the attentional stability measure.

The exploratory channel-wise analyses provided complementary information about the spatial distribution of suprathreshold responses. Several channels survived FDR correction in the younger and lower- or medium-motor-competence groups, whereas no channels survived correction in some older or higher-competence groups. Developmental neuroimaging research has proposed that cortical recruitment may shift from relatively diffuse to more selectively organized patterns with increasing age and task proficiency (Durston et al., 2006), and recent preschool fNIRS studies have similarly reported age- and task-related variation in cortical activation patterns (Du et al., 2025). However, because the present analyses did not include a direct measure of activation extent, spatial concentration, or focalization, the observed patterns cannot establish reduced neural recruitment or more selective cortical organization.

A neural-efficiency account therefore remains one possible interpretation, but it was not directly tested in the present study. Demonstrating neural efficiency would require explicit individual-level coupling between behavioral performance and neural activation, preferably together with measures of spatial concentration, functional connectivity, or network-level coordination. The present reliance on mean HbO responses, without complementary HbR analyses or connectivity measures, further limits mechanistic inference. Future studies should combine behavioral–neural coupling analyses with broader cortical and subcortical network measures to distinguish neural efficiency from task invariance, measurement insensitivity, and compensatory recruitment.

## 6 Conclusion

The present study found that higher motor competence was associated with greater task-based attentional stability in preschool children. The ROI models did not detect statistically significant coefficient differences in mean HbO responses across the reported age-group and motor-competence contrasts. The results therefore support a behavioral association between motor competence and attentional stability while placing clear limits on mechanistic interpretation. By combining behavioral assessment with child-friendly fNIRS, the study provides a foundation for future longitudinal and multimodal investigations of motor–attention–brain coupling during early childhood.

## CRediT authorship contribution statement

**Lulu Cheng:** Writing – review & editing, Funding acquisition. **Bingqi Fu:** Writing – original draft. **Haoran Mao:** Methodology. **Yang You:** Writing – original draft, Funding acquisition. **Yang Li:** Data curation. **Zeyu Sun:** Project administration.

## Funding

National Education Science Planning Project: “Construction of a Physical Ability Development Model and Co‑education Pathways for Preschool and Early School‑Age Children” (BLA220239); National Social Science Foundation of China: “AI-Empowered Precision Intervention and Dynamic Effect Evaluation for Pragmatic Impairment in Children with Autism” (25CYY083).

## Declaration of Competing Interest

The authors declare that they have no known competing financial interests or personal relationships that could have appeared to influence the work reported in this paper.

## Data Availability

Data will be made available on request.
