# Supplementary material for: Evaluating a neural-efficiency account of the association between motor competence and attentional stability in preschoolers: An fNIRS study
Source: Dev Cogn Neurosci. 2026 Jul 22;81:101787. doi: 10.1016/j.dcn.2026.101787 (PMC13499524; doi:10.1016/j.dcn.2026.101787)
Supplement: Supplementary file 1 [file mmc1.docx]

**Supplementary**

**Table S1. Normality Test Results of Attention Stability in Early, Medium, and Late Preschool Children**

| Early | | | | | |
| --- | --- | --- | --- | --- | --- |
| CH | S-D | statistic | degree of freedom | *p* | normality |
| 1 | S1-D1 | 0.87709 | 17 | 0.029756 | No |
| 2 | S1-D6 | 0.95427 | 17 | 0.44876 | Yes |
| 3 | S2-D2 | 0.96001 | 17 | 0.54345 | Yes |
| 4 | S2-D7 | 0.98959 | 17 | 0.99874 | Yes |
| 5 | S3-D2 | 0.91905 | 17 | 0.1423 | Yes |
| 6 | S3-D3 | 0.91538 | 17 | 0.11092 | Yes |
| 7 | S3-D8 | 0.91737 | 17 | 0.11914 | Yes |
| 8 | S4-D3 | 0.91865 | 17 | 0.12473 | Yes |
| 9 | S4-D4 | 0.98053 | 17 | 0.96175 | Yes |
| 10 | S4-D9 | 0.92236 | 17 | 0.14259 | Yes |
| 11 | S5-D4 | 0.96967 | 17 | 0.72571 | Yes |
| 12 | S5-D10 | 0.94252 | 17 | 0.29629 | Yes |
| 13 | S6-D5 | 0.7435 | 17 | 0.00071031 | No |
| 14 | S6-D11 | 0.85607 | 17 | 0.01329 | No |
| 15 | S7-D6 | 0.78713 | 17 | 0.0021218 | No |
| 16 | S7-D12 | 0.93631 | 17 | 0.27696 | Yes |
| 17 | S8-D2 | 0.76815 | 17 | 0.0013015 | No |
| 18 | S8-D7 | 0.97333 | 17 | 0.87372 | Yes |
| 19 | S8-D8 | 0.97264 | 17 | 0.78309 | Yes |
| 20 | S9-D3 | 0.96302 | 17 | 0.68879 | Yes |
| 21 | S9-D8 | 0.96099 | 17 | 0.65007 | Yes |
| 22 | S9-D9 | 0.93447 | 17 | 0.25835 | Yes |
| 23 | S10-D4 | 0.86874 | 17 | 0.022696 | No |
| 24 | S10-D9 | 0.97849 | 17 | 0.94213 | Yes |
| 25 | S10-D10 | 0.88213 | 17 | 0.035147 | No |
| 26 | S11-D5 | 0.95291 | 17 | 0.50418 | Yes |
| 27 | S11-D11 | 0.84021 | 17 | 0.0093827 | No |
| 28 | S12-D12 | 0.84517 | 17 | 0.01089 | No |
| 29 | S13-D11 | 0.91671 | 17 | 0.11634 | Yes |
| 30 | S13-D14 | 0.8656 | 17 | 0.020524 | No |
| 31 | S14-D11 | 0.93206 | 17 | 0.20276 | Yes |
| 32 | S14-D14 | 0.92086 | 17 | 0.13505 | Yes |
| 33 | S15-D14 | 0.94301 | 17 | 0.35569 | Yes |
| 34 | S16-D14 | 0.96181 | 17 | 0.66578 | Yes |
| 35 | S17-D1 | 0.88807 | 17 | 0.042879 | No |
| 36 | S17-D6 | 0.85733 | 17 | 0.015816 | No |
| 37 | S18-D6 | 0.95777 | 17 | 0.58996 | Yes |
| 38 | S18-D12 | 0.97702 | 17 | 0.92516 | Yes |
| 39 | S19-D13 | 0.94253 | 17 | 0.29641 | Yes |
| 40 | S20-D12 | 0.97028 | 17 | 0.82342 | Yes |
| 41 | S21-D13 | 0.76963 | 17 | 0.0013511 | No |
| 42 | S21-D15 | 0.94715 | 17 | 0.41298 | Yes |
| 43 | S21-D16 | 0.77805 | 17 | 0.0016752 | No |
| 44 | S22-D15 | 0.73119 | 17 | 0.00053079 | No |
| 45 | S23-D13 | 0.97072 | 17 | 0.83087 | Yes |
| 46 | S23-D16 | 0.84294 | 17 | 0.010179 | No |
| 47 | S24-D15 | 0.90592 | 17 | 0.079295 | Yes |
| 48 | S24-D16 | 0.77183 | 17 | 0.0014286 | No |

| Medium | | | | | |
| --- | --- | --- | --- | --- | --- |
| CH | S-D | statistic | degree of freedom | *p* | normality |
| 1 | S1-D1 | 0.93967 | 27 | 0.10876 | Yes |
| 2 | S1-D6 | 0.98 | 27 | 0.86243 | Yes |
| 3 | S2-D2 | 0.97424 | 27 | 0.71602 | Yes |
| 4 | S2-D7 | 0.98136 | 27 | 0.89163 | Yes |
| 5 | S3-D2 | 0.97256 | 27 | 0.57959 | Yes |
| 6 | S3-D3 | 0.9532 | 27 | 0.22038 | Yes |
| 7 | S3-D8 | 0.97045 | 27 | 0.52636 | Yes |
| 8 | S4-D3 | 0.92326 | 27 | 0.047324 | No |
| 9 | S4-D4 | 0.95545 | 27 | 0.2479 | Yes |
| 10 | S4-D9 | 0.85159 | 27 | 0.0020403 | No |
| 11 | S5-D4 | 0.97908 | 27 | 0.84097 | Yes |
| 12 | S5-D10 | 0.9599 | 27 | 0.3677 | Yes |
| 13 | S6-D5 | 0.90602 | 27 | 0.020671 | No |
| 14 | S6-D11 | 0.96836 | 27 | 0.47676 | Yes |
| 15 | S7-D6 | 0.84769 | 27 | 0.0017558 | No |
| 16 | S7-D12 | 0.7452 | 27 | 5.7668e-05 | No |
| 17 | S8-D2 | 0.948 | 27 | 0.16788 | Yes |
| 18 | S8-D7 | 0.97964 | 27 | 0.85411 | Yes |
| 19 | S8-D8 | 0.90411 | 27 | 0.018915 | No |
| 20 | S9-D3 | 0.92585 | 27 | 0.053844 | Yes |
| 21 | S9-D8 | 0.976 | 27 | 0.76325 | Yes |
| 22 | S9-D9 | 0.88893 | 27 | 0.0095332 | No |
| 23 | S10-D4 | 0.9554 | 27 | 0.28886 | Yes |
| 24 | S10-D9 | 0.97473 | 27 | 0.7293 | Yes |
| 25 | S10-D10 | 0.88493 | 27 | 0.0080055 | No |
| 26 | S11-D5 | 0.91119 | 27 | 0.026367 | No |
| 27 | S11-D11 | 0.83504 | 27 | 0.0010909 | No |
| 28 | S12-D12 | 0.98412 | 27 | 0.94112 | Yes |
| 29 | S13-D11 | 0.97679 | 27 | 0.69342 | Yes |
| 30 | S13-D14 | 0.92838 | 27 | 0.061115 | Yes |
| 31 | S14-D11 | 0.92264 | 27 | 0.045914 | No |
| 32 | S14-D14 | 0.91911 | 27 | 0.038622 | No |
| 33 | S15-D14 | 0.98192 | 27 | 0.90296 | Yes |
| 34 | S16-D14 | 0.96355 | 27 | 0.37601 | Yes |
| 35 | S17-D1 | 0.94009 | 27 | 0.11116 | Yes |
| 36 | S17-D6 | 0.97184 | 27 | 0.6509 | Yes |
| 37 | S18-D6 | 0.77826 | 27 | 0.00015737 | No |
| 38 | S18-D12 | 0.92135 | 27 | 0.0431 | No |
| 39 | S19-D13 | 0.97201 | 27 | 0.56546 | Yes |
| 40 | S20-D12 | 0.77321 | 27 | 0.00013433 | No |
| 41 | S21-D13 | 0.93322 | 27 | 0.078082 | Yes |
| 42 | S21-D15 | 0.93624 | 27 | 0.091137 | Yes |
| 43 | S21-D16 | 0.90394 | 27 | 0.018768 | No |
| 44 | S22-D15 | 0.97434 | 27 | 0.71879 | Yes |
| 45 | S23-D13 | 0.93656 | 27 | 0.092639 | Yes |
| 46 | S23-D16 | 0.89158 | 27 | 0.010719 | No |
| 47 | S24-D15 | 0.95211 | 27 | 0.20814 | Yes |
| 48 | S24-D16 | 0.968 | 27 | 0.46859 | Yes |

| Late | | | | | |
| --- | --- | --- | --- | --- | --- |
| CH | S-D | statistic | degree of freedom | *p* | normality |
| 1 | S1-D1 | 0.93503 | 18 | 0.23782 | Yes |
| 2 | S1-D6 | 0.90968 | 18 | 0.079148 | Yes |
| 3 | S2-D2 | 0.91687 | 18 | 0.10326 | Yes |
| 4 | S2-D7 | 0.92857 | 18 | 0.18323 | Yes |
| 5 | S3-D2 | 0.97915 | 18 | 0.94082 | Yes |
| 6 | S3-D3 | 0.98039 | 18 | 0.95397 | Yes |
| 7 | S3-D8 | 0.91126 | 18 | 0.08388 | Yes |
| 8 | S4-D3 | 0.89955 | 18 | 0.056428 | Yes |
| 9 | S4-D4 | 0.87975 | 18 | 0.027405 | No |
| 10 | S4-D9 | 0.96516 | 18 | 0.70341 | Yes |
| 11 | S5-D4 | 0.91118 | 18 | 0.083643 | Yes |
| 12 | S5-D10 | 0.9418 | 18 | 0.311 | Yes |
| 13 | S6-D5 | 0.79371 | 18 | 0.0019796 | No |
| 14 | S6-D11 | 0.89542 | 18 | 0.047276 | No |
| 15 | S7-D6 | 0.78837 | 18 | 0.0017119 | No |
| 16 | S7-D12 | 0.98364 | 18 | 0.9797 | Yes |
| 17 | S8-D2 | 0.95596 | 18 | 0.5259 | Yes |
| 18 | S8-D7 | 0.97171 | 18 | 0.82908 | Yes |
| 19 | S8-D8 | 0.96771 | 18 | 0.75375 | Yes |
| 20 | S9-D3 | 0.92564 | 18 | 0.16265 | Yes |
| 21 | S9-D8 | 0.924 | 18 | 0.13489 | Yes |
| 22 | S9-D9 | 0.95475 | 18 | 0.4285 | Yes |
| 23 | S10-D4 | 0.59486 | 18 | 2.2217e-05 | No |
| 24 | S10-D9 | 0.52485 | 18 | 6.3002e-06 | No |
| 25 | S10-D10 | 0.60802 | 18 | 2.8571e-05 | No |
| 26 | S11-D5 | 0.86404 | 18 | 0.016218 | No |
| 27 | S11-D11 | 0.91401 | 18 | 0.10128 | Yes |
| 28 | S12-D12 | 0.96647 | 18 | 0.72923 | Yes |
| 29 | S13-D11 | 0.94155 | 18 | 0.26236 | Yes |
| 30 | S13-D14 | 0.96578 | 18 | 0.71563 | Yes |
| 31 | S14-D11 | 0.80742 | 18 | 0.0028994 | No |
| 32 | S14-D14 | 0.7822 | 18 | 0.0014506 | No |
| 33 | S15-D14 | 0.89453 | 18 | 0.045805 | No |
| 34 | S16-D14 | 0.95136 | 18 | 0.37878 | Yes |
| 35 | S17-D1 | 0.97303 | 18 | 0.85237 | Yes |
| 36 | S17-D6 | 0.46343 | 18 | 2.2877e-06 | No |
| 37 | S18-D6 | 0.8983 | 18 | 0.05238 | Yes |
| 38 | S18-D12 | 0.9596 | 18 | 0.59386 | Yes |
| 39 | S19-D13 | 0.94468 | 18 | 0.34775 | Yes |
| 40 | S20-D12 | 0.90466 | 18 | 0.065877 | Yes |
| 41 | S21-D13 | 0.98497 | 18 | 0.98667 | Yes |
| 42 | S21-D15 | 0.90881 | 18 | 0.076674 | Yes |
| 43 | S21-D16 | 0.75261 | 18 | 0.00067565 | No |
| 44 | S22-D15 | 0.94004 | 18 | 0.24785 | Yes |
| 45 | S23-D13 | 0.7986 | 18 | 0.0022652 | No |
| 46 | S23-D16 | 0.82568 | 18 | 0.0049174 | No |
| 47 | S24-D15 | 0.96377 | 18 | 0.58539 | Yes |
| 48 | S24-D16 | 0.84906 | 18 | 0.010032 | No |

**Table S2. Homogeneity Results of Attention Stability in Early, Medium, and Late Preschool Children**

| CH | S-D | statistic | df1 | df2 | *p* | Homogeneity |
| --- | --- | --- | --- | --- | --- | --- |
| 1 | S1-D1 | 0.45342 | 2 | 59 | 0.63765 | Yes |
| 2 | S1-D6 | 0.067968 | 2 | 59 | 0.93436 | Yes |
| 3 | S2-D2 | 0.9406 | 2 | 59 | 0.39617 | Yes |
| 4 | S2-D7 | 2.7651 | 2 | 59 | 0.071139 | Yes |
| 5 | S3-D2 | 2.3975 | 2 | 59 | 0.099752 | Yes |
| 6 | S3-D3 | 3.6596 | 2 | 59 | 0.031754 | No |
| 7 | S3-D8 | 1.6162 | 2 | 59 | 0.20733 | Yes |
| 8 | S4-D3 | 2.8169 | 2 | 59 | 0.067856 | Yes |
| 9 | S4-D4 | 0.34432 | 2 | 59 | 0.71012 | Yes |
| 10 | S4-D9 | 0.50534 | 2 | 59 | 0.60589 | Yes |
| 11 | S5-D4 | 2.0885 | 2 | 59 | 0.13294 | Yes |
| 12 | S5-D10 | 3.4022 | 2 | 59 | 0.039959 | No |
| 13 | S6-D5 | 0.64436 | 2 | 59 | 0.52865 | Yes |
| 14 | S6-D11 | 0.20029 | 2 | 59 | 0.81904 | Yes |
| 15 | S7-D6 | 1.6325 | 2 | 59 | 0.20415 | Yes |
| 16 | S7-D12 | 0.0038754 | 2 | 59 | 0.99613 | Yes |
| 17 | S8-D2 | 1.5565 | 2 | 59 | 0.2194 | Yes |
| 18 | S8-D7 | 0.17002 | 2 | 59 | 0.84406 | Yes |
| 19 | S8-D8 | 1.9363 | 2 | 59 | 0.1533 | Yes |
| 20 | S9-D3 | 5.0725 | 2 | 59 | 0.0092725 | No |
| 21 | S9-D8 | 5.1211 | 2 | 59 | 0.0088963 | No |
| 22 | S9-D9 | 0.22755 | 2 | 59 | 0.79718 | Yes |
| 23 | S10-D4 | 0.38817 | 2 | 59 | 0.68002 | Yes |
| 24 | S10-D9 | 0.27253 | 2 | 59 | 0.7624 | Yes |
| 25 | S10-D10 | 0.0088312 | 2 | 59 | 0.99121 | Yes |
| 26 | S11-D5 | 0.79766 | 2 | 59 | 0.45518 | Yes |
| 27 | S11-D11 | 0.17484 | 2 | 59 | 0.84002 | Yes |
| 28 | S12-D12 | 3.6083 | 2 | 59 | 0.033237 | No |
| 29 | S13-D11 | 0.64431 | 2 | 59 | 0.52868 | Yes |
| 30 | S13-D14 | 0.15398 | 2 | 59 | 0.85763 | Yes |
| 31 | S14-D11 | 3.3543 | 2 | 59 | 0.041714 | No |
| 32 | S14-D14 | 1.4106 | 2 | 59 | 0.2521 | Yes |
| 33 | S15-D14 | 1.0117 | 2 | 59 | 0.36981 | Yes |
| 34 | S16-D14 | 0.32178 | 2 | 59 | 0.72612 | Yes |
| 35 | S17-D1 | 0.95247 | 2 | 59 | 0.39164 | Yes |
| 36 | S17-D6 | 0.50639 | 2 | 59 | 0.60526 | Yes |
| 37 | S18-D6 | 2.0691 | 2 | 59 | 0.13537 | Yes |
| 38 | S18-D12 | 0.57488 | 2 | 59 | 0.56589 | Yes |
| 39 | S19-D13 | 0.70839 | 2 | 59 | 0.49658 | Yes |
| 40 | S20-D12 | 1.6033 | 2 | 59 | 0.20987 | Yes |
| 41 | S21-D13 | 0.19431 | 2 | 59 | 0.82393 | Yes |
| 42 | S21-D15 | 1.1422 | 2 | 59 | 0.32606 | Yes |
| 43 | S21-D16 | 1.1955 | 2 | 59 | 0.30979 | Yes |
| 44 | S22-D15 | 1.583 | 2 | 59 | 0.21396 | Yes |
| 45 | S23-D13 | 2.1967 | 2 | 59 | 0.12019 | Yes |
| 46 | S23-D16 | 0.027559 | 2 | 59 | 0.97283 | Yes |
| 47 | S24-D15 | 0.24584 | 2 | 59 | 0.78284 | Yes |
| 48 | S24-D16 | 0.23255 | 2 | 59 | 0.79323 | Yes |

**Table S3. Normality Test Results of Attention Stability in Low, Medium, and High Motor Development Ability of Preschool Children**

| Low | | | | | |
| --- | --- | --- | --- | --- | --- |
| CH | S-D | statistic | degree of freedom | *p* | normality |
| 1 | S1-D1 | 0.92808 | 16 | 0.22729 | Yes |
| 2 | S1-D6 | 0.89506 | 16 | 0.063464 | Yes |
| 3 | S2-D2 | 0.84874 | 16 | 0.014867 | No |
| 4 | S2-D7 | 0.9495 | 16 | 0.48183 | Yes |
| 5 | S3-D2 | 0.95577 | 16 | 0.58617 | Yes |
| 6 | S3-D3 | 0.92838 | 16 | 0.19764 | Yes |
| 7 | S3-D8 | 0.91097 | 16 | 0.12063 | Yes |
| 8 | S4-D3 | 0.82337 | 16 | 0.007182 | No |
| 9 | S4-D4 | 0.98612 | 16 | 0.99413 | Yes |
| 10 | S4-D9 | 0.96567 | 16 | 0.76454 | Yes |
| 11 | S5-D4 | 0.94349 | 16 | 0.33337 | Yes |
| 12 | S5-D10 | 0.94962 | 16 | 0.48365 | Yes |
| 13 | S6-D5 | 0.91904 | 16 | 0.14293 | Yes |
| 14 | S6-D11 | 0.9114 | 16 | 0.10996 | Yes |
| 15 | S7-D6 | 0.93565 | 16 | 0.29907 | Yes |
| 16 | S7-D12 | 0.95416 | 16 | 0.47591 | Yes |
| 17 | S8-D2 | 0.95189 | 16 | 0.44207 | Yes |
| 18 | S8-D7 | 0.93201 | 16 | 0.26225 | Yes |
| 19 | S8-D8 | 0.97449 | 16 | 0.90505 | Yes |
| 20 | S9-D3 | 0.98008 | 16 | 0.9642 | Yes |
| 21 | S9-D8 | 0.92999 | 16 | 0.20897 | Yes |
| 22 | S9-D9 | 0.86139 | 16 | 0.020129 | No |
| 23 | S10-D4 | 0.95222 | 16 | 0.52558 | Yes |
| 24 | S10-D9 | 0.93243 | 16 | 0.22751 | Yes |
| 25 | S10-D10 | 0.81817 | 16 | 0.0062206 | No |
| 26 | S11-D5 | 0.95896 | 16 | 0.55398 | Yes |
| 27 | S11-D11 | 0.70816 | 16 | 0.00042346 | No |
| 28 | S12-D12 | 0.93712 | 16 | 0.26773 | Yes |
| 29 | S13-D11 | 0.90236 | 16 | 0.087692 | Yes |
| 30 | S13-D14 | 0.95732 | 16 | 0.61346 | Yes |
| 31 | S14-D11 | 0.97084 | 16 | 0.852 | Yes |
| 32 | S14-D14 | 0.7748 | 16 | 0.0020007 | No |
| 33 | S15-D14 | 0.95395 | 16 | 0.47273 | Yes |
| 34 | S16-D14 | 0.89346 | 16 | 0.060186 | Yes |
| 35 | S17-D1 | 0.974 | 16 | 0.89843 | Yes |
| 36 | S17-D6 | 0.92921 | 16 | 0.23683 | Yes |
| 37 | S18-D6 | 0.76678 | 16 | 0.0016408 | No |
| 38 | S18-D12 | 0.87393 | 16 | 0.032072 | No |
| 39 | S19-D13 | 0.88774 | 16 | 0.0513 | Yes |
| 40 | S20-D12 | 0.79662 | 16 | 0.0034914 | No |
| 41 | S21-D13 | 0.96054 | 16 | 0.67155 | Yes |
| 42 | S21-D15 | 0.94013 | 16 | 0.35058 | Yes |
| 43 | S21-D16 | 0.78262 | 16 | 0.002435 | No |
| 44 | S22-D15 | 0.62827 | 16 | 8.32E-05 | No |
| 45 | S23-D13 | 0.91623 | 16 | 0.12975 | Yes |
| 46 | S23-D16 | 0.95912 | 16 | 0.64588 | Yes |
| 47 | S24-D15 | 0.95459 | 16 | 0.48259 | Yes |
| 48 | S24-D16 | 0.90495 | 16 | 0.096511 | Yes |
|  | | | | | |
| Medium | | | | | |
| CH | S-D | statistic | degree of freedom | *p* | normality |
| 1 | S1-D1 | 0.90291 | 28 | 0.015678 | No |
| 2 | S1-D6 | 0.94362 | 28 | 0.12281 | Yes |
| 3 | S2-D2 | 0.98157 | 28 | 0.88615 | Yes |
| 4 | S2-D7 | 0.97509 | 28 | 0.72124 | Yes |
| 5 | S3-D2 | 0.89593 | 28 | 0.011331 | No |
| 6 | S3-D3 | 0.96841 | 28 | 0.53854 | Yes |
| 7 | S3-D8 | 0.93575 | 28 | 0.080863 | Yes |
| 8 | S4-D3 | 0.96616 | 28 | 0.48226 | Yes |
| 9 | S4-D4 | 0.9208 | 28 | 0.037452 | No |
| 10 | S4-D9 | 0.97975 | 28 | 0.84431 | Yes |
| 11 | S5-D4 | 0.91675 | 28 | 0.030602 | No |
| 12 | S5-D10 | 0.95362 | 28 | 0.21044 | Yes |
| 13 | S6-D5 | 0.90643 | 28 | 0.018525 | No |
| 14 | S6-D11 | 0.93516 | 28 | 0.078404 | Yes |
| 15 | S7-D6 | 0.67198 | 28 | 6.11E-06 | No |
| 16 | S7-D12 | 0.9453 | 28 | 0.13442 | Yes |
| 17 | S8-D2 | 0.82598 | 28 | 0.00064744 | No |
| 18 | S8-D7 | 0.98372 | 28 | 0.9281 | Yes |
| 19 | S8-D8 | 0.88858 | 28 | 0.0081165 | No |
| 20 | S9-D3 | 0.89969 | 28 | 0.013481 | No |
| 21 | S9-D8 | 0.96932 | 28 | 0.5626 | Yes |
| 22 | S9-D9 | 0.90684 | 28 | 0.016608 | No |
| 23 | S10-D4 | 0.73817 | 28 | 3.71E-05 | No |
| 24 | S10-D9 | 0.75036 | 28 | 5.32E-05 | No |
| 25 | S10-D10 | 0.86236 | 28 | 0.0026417 | No |
| 26 | S11-D5 | 0.94939 | 28 | 0.16758 | Yes |
| 27 | S11-D11 | 0.93073 | 28 | 0.064313 | Yes |
| 28 | S12-D12 | 0.95814 | 28 | 0.31473 | Yes |
| 29 | S13-D11 | 0.95718 | 28 | 0.25489 | Yes |
| 30 | S13-D14 | 0.92292 | 28 | 0.041692 | No |
| 31 | S14-D11 | 0.81988 | 28 | 0.00051913 | No |
| 32 | S14-D14 | 0.8659 | 28 | 0.0030565 | No |
| 33 | S15-D14 | 0.96309 | 28 | 0.34904 | Yes |
| 34 | S16-D14 | 0.96336 | 28 | 0.41757 | Yes |
| 35 | S17-D1 | 0.82143 | 28 | 0.00054892 | No |
| 36 | S17-D6 | 0.62034 | 28 | 1.74E-06 | No |
| 37 | S18-D6 | 0.92049 | 28 | 0.036884 | No |
| 38 | S18-D12 | 0.96537 | 28 | 0.393 | Yes |
| 39 | S19-D13 | 0.97657 | 28 | 0.76192 | Yes |
| 40 | S20-D12 | 0.90924 | 28 | 0.021205 | No |
| 41 | S21-D13 | 0.84366 | 28 | 0.0012572 | No |
| 42 | S21-D15 | 0.86985 | 28 | 0.0036031 | No |
| 43 | S21-D16 | 0.76837 | 28 | 9.25E-05 | No |
| 44 | S22-D15 | 0.97285 | 28 | 0.65864 | Yes |
| 45 | S23-D13 | 0.95611 | 28 | 0.24067 | Yes |
| 46 | S23-D16 | 0.91287 | 28 | 0.025289 | No |
| 47 | S24-D15 | 0.9476 | 28 | 0.15215 | Yes |
| 48 | S24-D16 | 0.82646 | 28 | 0.00065888 | No |

| High | | | | | |
| --- | --- | --- | --- | --- | --- |
| CH | S-D | statistic | degree of freedom | *p* | normality |
| 1 | S1-D1 | 0.94277 | 17 | 0.35255 | Yes |
| 2 | S1-D6 | 0.96991 | 17 | 0.81686 | Yes |
| 3 | S2-D2 | 0.97143 | 17 | 0.75989 | Yes |
| 4 | S2-D7 | 0.96166 | 17 | 0.6629 | Yes |
| 5 | S3-D2 | 0.93292 | 17 | 0.24346 | Yes |
| 6 | S3-D3 | 0.87024 | 17 | 0.023816 | No |
| 7 | S3-D8 | 0.95125 | 17 | 0.40425 | Yes |
| 8 | S4-D3 | 0.88347 | 17 | 0.036756 | No |
| 9 | S4-D4 | 0.9679 | 17 | 0.78063 | Yes |
| 10 | S4-D9 | 0.86928 | 17 | 0.023094 | No |
| 11 | S5-D4 | 0.94071 | 17 | 0.32673 | Yes |
| 12 | S5-D10 | 0.97744 | 17 | 0.93033 | Yes |
| 13 | S6-D5 | 0.83082 | 17 | 0.0071103 | No |
| 14 | S6-D11 | 0.7936 | 17 | 0.0025185 | No |
| 15 | S7-D6 | 0.9244 | 17 | 0.15352 | Yes |
| 16 | S7-D12 | 0.81851 | 17 | 0.0049936 | No |
| 17 | S8-D2 | 0.94108 | 17 | 0.3312 | Yes |
| 18 | S8-D7 | 0.96339 | 17 | 0.69576 | Yes |
| 19 | S8-D8 | 0.9692 | 17 | 0.80433 | Yes |
| 20 | S9-D3 | 0.93905 | 17 | 0.30714 | Yes |
| 21 | S9-D8 | 0.95614 | 17 | 0.56047 | Yes |
| 22 | S9-D9 | 0.9596 | 17 | 0.62402 | Yes |
| 23 | S10-D4 | 0.94947 | 17 | 0.3797 | Yes |
| 24 | S10-D9 | 0.92841 | 17 | 0.20469 | Yes |
| 25 | S10-D10 | 0.96425 | 17 | 0.71213 | Yes |
| 26 | S11-D5 | 0.92542 | 17 | 0.18234 | Yes |
| 27 | S11-D11 | 0.96665 | 17 | 0.75759 | Yes |
| 28 | S12-D12 | 0.9601 | 17 | 0.54503 | Yes |
| 29 | S13-D11 | 0.97099 | 17 | 0.83555 | Yes |
| 30 | S13-D14 | 0.94578 | 17 | 0.39325 | Yes |
| 31 | S14-D11 | 0.93482 | 17 | 0.22413 | Yes |
| 32 | S14-D14 | 0.97032 | 17 | 0.82395 | Yes |
| 33 | S15-D14 | 0.94245 | 17 | 0.34845 | Yes |
| 34 | S16-D14 | 0.9671 | 17 | 0.766 | Yes |
| 35 | S17-D1 | 0.97042 | 17 | 0.82572 | Yes |
| 36 | S17-D6 | 0.88354 | 17 | 0.036297 | No |
| 37 | S18-D6 | 0.9571 | 17 | 0.57773 | Yes |
| 38 | S18-D12 | 0.97389 | 17 | 0.80679 | Yes |
| 39 | S19-D13 | 0.96457 | 17 | 0.62708 | Yes |
| 40 | S20-D12 | 0.67145 | 17 | 0.00014172 | No |
| 41 | S21-D13 | 0.99355 | 17 | 0.99996 | Yes |
| 42 | S21-D15 | 0.96542 | 17 | 0.73434 | Yes |
| 43 | S21-D16 | 0.84655 | 17 | 0.011352 | No |
| 44 | S22-D15 | 0.95239 | 17 | 0.49544 | Yes |
| 45 | S23-D13 | 0.92682 | 17 | 0.16761 | Yes |
| 46 | S23-D16 | 0.86717 | 17 | 0.021584 | No |
| 47 | S24-D15 | 0.94863 | 17 | 0.43523 | Yes |
| 48 | S24-D16 | 0.90761 | 17 | 0.084157 | Yes |

**Table S4. Homogeneity Results of Attention Stability in Low, Medium, and High Motor Development Ability of Preschool Children**

| CH | S-D | statistic | df1 | df2 | *p* | Homogeneity |
| --- | --- | --- | --- | --- | --- | --- |
| 1 | S1-D1 | 6.9605 | 2 | 58 | 0.0019525 | No |
| 2 | S1-D6 | 1.7505 | 2 | 58 | 0.18273 | Yes |
| 3 | S2-D2 | 0.54947 | 2 | 58 | 0.58023 | Yes |
| 4 | S2-D7 | 0.6573 | 2 | 58 | 0.52207 | Yes |
| 5 | S3-D2 | 1.7542 | 2 | 58 | 0.18211 | Yes |
| 6 | S3-D3 | 1.6854 | 2 | 58 | 0.19432 | Yes |
| 7 | S3-D8 | 1.4914 | 2 | 58 | 0.23355 | Yes |
| 8 | S4-D3 | 0.042474 | 2 | 58 | 0.95845 | Yes |
| 9 | S4-D4 | 1.2669 | 2 | 58 | 0.28937 | Yes |
| 10 | S4-D9 | 2.7385 | 2 | 58 | 0.073038 | Yes |
| 11 | S5-D4 | 0.76194 | 2 | 58 | 0.47137 | Yes |
| 12 | S5-D10 | 1.0439 | 2 | 58 | 0.35861 | Yes |
| 13 | S6-D5 | 1.4398 | 2 | 58 | 0.24532 | Yes |
| 14 | S6-D11 | 0.99069 | 2 | 58 | 0.37752 | Yes |
| 15 | S7-D6 | 0.58467 | 2 | 58 | 0.56054 | Yes |
| 16 | S7-D12 | 2.5146 | 2 | 58 | 0.089677 | Yes |
| 17 | S8-D2 | 1.3068 | 2 | 58 | 0.27854 | Yes |
| 18 | S8-D7 | 1.8772 | 2 | 58 | 0.1622 | Yes |
| 19 | S8-D8 | 3.0148 | 2 | 58 | 0.056802 | Yes |
| 20 | S9-D3 | 0.15722 | 2 | 58 | 0.85488 | Yes |
| 21 | S9-D8 | 0.36136 | 2 | 58 | 0.69828 | Yes |
| 22 | S9-D9 | 0.90035 | 2 | 58 | 0.41203 | Yes |
| 23 | S10-D4 | 2.0261 | 2 | 58 | 0.14108 | Yes |
| 24 | S10-D9 | 2.5645 | 2 | 58 | 0.085658 | Yes |
| 25 | S10-D10 | 2.9658 | 2 | 58 | 0.059384 | Yes |
| 26 | S11-D5 | 4.4807 | 2 | 58 | 0.015506 | No |
| 27 | S11-D11 | 1.8187 | 2 | 58 | 0.17136 | Yes |
| 28 | S12-D12 | 2.3306 | 2 | 58 | 0.10628 | Yes |
| 29 | S13-D11 | 0.7412 | 2 | 58 | 0.481 | Yes |
| 30 | S13-D14 | 0.56743 | 2 | 58 | 0.57009 | Yes |
| 31 | S14-D11 | 0.72294 | 2 | 58 | 0.48965 | Yes |
| 32 | S14-D14 | 0.124 | 2 | 58 | 0.88362 | Yes |
| 33 | S15-D14 | 0.95713 | 2 | 58 | 0.38997 | Yes |
| 34 | S16-D14 | 1.3644 | 2 | 58 | 0.26361 | Yes |
| 35 | S17-D1 | 1.2837 | 2 | 58 | 0.28477 | Yes |
| 36 | S17-D6 | 0.67543 | 2 | 58 | 0.5129 | Yes |
| 37 | S18-D6 | 0.75399 | 2 | 58 | 0.47504 | Yes |
| 38 | S18-D12 | 0.26637 | 2 | 58 | 0.76708 | Yes |
| 39 | S19-D13 | 0.31988 | 2 | 58 | 0.72751 | Yes |
| 40 | S20-D12 | 1.9886 | 2 | 58 | 0.14611 | Yes |
| 41 | S21-D13 | 0.77536 | 2 | 58 | 0.46525 | Yes |
| 42 | S21-D15 | 0.38166 | 2 | 58 | 0.68443 | Yes |
| 43 | S21-D16 | 0.54825 | 2 | 58 | 0.58093 | Yes |
| 44 | S22-D15 | 0.86986 | 2 | 58 | 0.42441 | Yes |
| 45 | S23-D13 | 0.774 | 2 | 58 | 0.46587 | Yes |
| 46 | S23-D16 | 2.8342 | 2 | 58 | 0.066932 | Yes |
| 47 | S24-D15 | 0.26162 | 2 | 58 | 0.77071 | Yes |
| 48 | S24-D16 | 1.8475 | 2 | 58 | 0.16679 | Yes |

**Table S5A. Brain Regions with Differences in Oxygenated Hemoglobin Concentration in Early Preschool Children**

| CH | S-D | T | degree of freedom | *p* | significant | Putative cortical region |
| --- | --- | --- | --- | --- | --- | --- |
| 28 | S12-D12 | 3.7239 | 16 | .044 | Yes | Putative premotor/supplementary motor region |
| 34 | S16-D14 | 4.7101 | 16 | .011 | Yes | Putative premotor/supplementary motor region |

**Table S5B. Brain Regions with Differences in Oxygenated Hemoglobin Concentration in Medium Preschool Children**

| CH | S-D | T | degree of freedom | *p* | significant | Putative cortical region |
| --- | --- | --- | --- | --- | --- | --- |
| 3 | S2-D2 | 2.8797 | 26 | .034 | Yes | Dorsolateral prefrontal cortex |
| 4 | S2-D7 | 2.7529 | 26 | .039 | Yes | pars triangularis Putative inferior frontal region |
| 5 | S3-D2 | 3.4765 | 26 | 0.013 | Yes | Orbitofrontal area |
| 6 | S3-D3 | 3.819 | 26 | .013 | Yes | Orbitofrontal area |
| 7 | S3-D8 | 3.6619 | 26 | .013 | Yes | Frontopolar area |
| 17 | S8-D2 | 3.1496 | 26 | .022 | Yes | Dorsolateral prefrontal cortex |
| 18 | S8-D7 | 3.2187 | 26 | .021 | Yes | pars triangularis Putative inferior frontal region |
| 19 | S8-D8 | 3.548 | 26 | .013 | Yes | Dorsolateral prefrontal cortex |
| 21 | S9-D8 | 2.8087 | 26 | .037 | Yes | Frontopolar area |
| 28 | S12-D12 | 2.9986 | 26 | .028 | Yes | Putative premotor/supplementary motor region |
| 29 | S13-D11 | 3.7514 | 26 | .013 | Yes | Putative motor-related region |
| 30 | S13-D14 | 3.4555 | 26 | .013 | Yes | Putative motor-related region |
| 33 | S15-D14 | 3.4588 | 26 | .013 | Yes | Putative motor-related region |

**Table S6A. Brain Regions with Differences in Oxygenated Hemoglobin Concentration in Low Motor Ability of Children**

| CH | S-D | T | degree of freedom | *p* | significant | Putative cortical region |
| --- | --- | --- | --- | --- | --- | --- |
| 7 | S3-D8 | 3.8803 | 15 | .024 | Yes | Frontopolar area |
| 17 | S8-D2 | 5.0505 | 15 | .003 | Yes | Dorsolateral prefrontal cortex |
| 18 | S8-D7 | 3.4901 | 15 | .039 | Yes | pars triangularis Putative inferior frontal region |
| 34 | S16-D14 | 5.2831 | 15 | .003 | Yes | Putative premotor/supplementary motor region |

**Table S6B. Brain Regions with Differences in Oxygenated Hemoglobin Concentration in Medium Motor Ability of Children**

| CH | S-D | T | degree of freedom | *p* | significant | Putative cortical region |
| --- | --- | --- | --- | --- | --- | --- |
| 4 | S2-D7 | 2.8654 | 27 | .043 | Yes | pars triangularis Putative inferior frontal region |
| 5 | S3-D2 | 4.4239 | 27 | .003 | Yes | Orbitofrontal area |
| 6 | S3-D3 | 3.2914 | 27 | .033 | Yes | Orbitofrontal area |
| 19 | S8-D8 | 3.0997 | 27 | .036 | Yes | Dorsolateral prefrontal cortex |
| 22 | S9-D9 | -2.9488 | 27 | .043 | Yes | Frontopolar area |
| 25 | S10-D10 | -3.5845 | 27 | .021 | Yes | pars triangularis Putative inferior frontal region |
| 33 | S15-D14 | 2.8813 | 27 | .043 | Yes | Putative motor-related region |
| 34 | S16-D14 | 4.7618 | 27 | .003 | Yes | Putative premotor/supplementary motor region |
| 48 | S24-D16 | 3.1562 | 27 | .036 | Yes | Putative posterior visual-related region |

**Table S7A. Results of the Variance Analysis for Children with Low and Medium Motor Development Levels**

| CH | S-D | (I) | (J) | Mean Difference(I-J) | 95% CI Lower Bound | 95% CI Upper Bound | *p* | Putative cortical region |
| --- | --- | --- | --- | --- | --- | --- | --- | --- |
| 25 | S10-D10 | low | medium | .165 | .029 | .300 | .018 | pars triangularis Putative inferior frontal region |
| 48 | S24-D16 | low | medium | -.129 | -.240 | -.017 | .024 | Putative posterior visual-related region |

**Table S7B. Results of the Variance Analysis for Children with Medium and High Motor Development Levels**

| CH | S-D | (I) | (J) | Mean Difference(I-J) | 95% CI Lower Bound | 95% CI Upper Bound | *p* | Putative cortical region |
| --- | --- | --- | --- | --- | --- | --- | --- | --- |
| 25 | S10-D10 | medium | high | -.141 | -.274 | -.009 | .037 | pars triangularis Putative inferior frontal region |
| 34 | S16-D14 | medium | high | .164 | .057 | .271 | .003 | Putative premotor/supplementary motor region |
| 42 | S21-D15 | medium | high | .097 | .018 | .177 | .017 | Putative posterior visual-related region |

**Table S7C. Results of the Variance Analysis for Children with Low and High Motor Development Levels**

| CH | S-D | (I) | (J) | Mean Difference(I-J) | 95% CI Lower Bound | 95% CI Upper Bound | *p* | Putative cortical region |
| --- | --- | --- | --- | --- | --- | --- | --- | --- |
| 34 | S16-D14 | low | high | .202 | .081 | .323 | .002 | pars triangularis Putative inferior frontal region |
| 40 | S20-D12 | low | high | -.119 | -.225 | -.013 | .029 | Putative motor-related region |
